# SARS-CoV-2 prevalence associated to low socioeconomic status and overcrowding in an LMIC megacity: A population-based seroepidemiological survey in Lima, Peru

**DOI:** 10.1016/j.eclinm.2021.100801

**Published:** 2021-03-30

**Authors:** Mary F. Reyes-Vega, M.Gabriela Soto-Cabezas, Fany Cárdenas, Kevin S. Martel, Andree Valle, Juan Valverde, Margot Vidal-Anzardo, María Elena Falcón, César V. Munayco, Peru COVID-19 Working Group

**Affiliations:** a Centro Nacional de Epidemiología, Prevención y Control de Enfermedades, Peruvian Ministry of Health, Jr. Daniel Olaechea N°. 199, Jesús María, Lima, Peru; b Instituto Nacional de Salud, Peruvian Ministry of Health, Av. Defensores del Morro 2268, Chorrillos, Lima, Peru; c Instituto Nacional de Estadística e Informática, Av. Gral. Garzón 654 - 658, Jesús María, Lima, Peru

**Keywords:** SARS-CoV-2, COVID-19, Prevalence, Seroprevalence, Population-based, Peru, Lima

## Abstract

**Background:**

Worldwide, Peru has one of the highest infection fatality rates of COVID-19, and its capital city, Lima, accumulates roughly 50% of diagnosed cases. Despite surveillance efforts to assess the extent of the pandemic, reported cases and deaths only capture a fraction of its impact due to COVID-19′s broad clinical spectrum. This study aimed to estimate the seroprevalence of SARS-CoV-2 in Lima, stratified by age, sex, region, socioeconomic status (SES), overcrowding, and symptoms.

**Methods:**

We conducted a multi-stage, population-based serosurvey in Lima, between June 28th and July 9th, 2020, after 115 days of the index case and after the first peak cases. We collected whole blood samples by finger-prick and applied a structured questionnaire. A point-of-care rapid serological test assessed IgM and IgG antibodies against SARS-CoV-2. Seroprevalence estimates were adjusted by sampling weights and test performance. Additionally, we performed RT-PCR molecular assays to seronegatives and estimated the infection prevalence.

**Findings:**

We enrolled 3212 participants from 797 households and 241 sample clusters from Lima in the analysis. The SARS-CoV-2 seroprevalence was 20·8% (95%CI 17·2–23·5), and the prevalence was 25·2% (95%CI 22·5–28·2). Seroprevalence was equally distributed by sex (aPR=0·96 [95%CI 0·85–1·09, *p* = 0·547]) and across all age groups, including ≥60 versus ≤11 years old (aPR=0·96 [95%CI 0·73–1·27, *p* = 0·783]). A gradual decrease in SES was associated with higher seroprevalence (aPR=3·41 [95%CI 1·90–6·12, *p*<0·001] in low SES). Also, a gradual increase in the overcrowding index was associated with higher seroprevalence (aPR=1·99 [95%CI 1·41–2·81, *p*<0·001] in the fourth quartile). Seroprevalence was also associated with contact with a suspected or confirmed COVID-19 case, whether a household member (48·9%, aPR=2·67 [95%CI 2·06–3·47, *p*<0·001]), other family members (27·3%, aPR=1·66 [95%CI 1·15–2·40, *p* = 0·008]) or a workmate (34·1%, aPR=2·26 [95%CI 1·53–3·35, *p*<0·001]). More than half of seropositive participants reported never having had symptoms (56·1%, 95% CI 49·7–62·3).

**Interpretation:**

This first estimate of SARS-CoV-2 seroprevalence in Lima shows an intense transmission scenario, despite the government's numerous interventions early established. Susceptibles across age groups show that physical distancing interventions must not be relaxed. SES and overcrowding households are associated with seroprevalence. This study highlights the importance of considering the existing social inequalities for implementing the response to control transmission in low- and middle-income countries.

**Research in context**

**Evidence before this study**

Worldwide, Peru has one of the highest infection fatality rates of COVID-19, and its capital city, Lima, accumulates roughly 50% of diagnosed cases in the country. We searched PubMed, Scielo, and medRxiv preprint server for papers in any language, published from November 1st, 2019 onwards, for epidemiological studies of the prevalence of SARS-CoV-2 infection in Peru and other low-and middle-income countries (LMIC). There are no published studies of seroprevalence of SARS-CoV-2 conducted in Lima or other regions of Peru. In Latin America, only Brazil has published population-based reports. Few population-based studies on the prevalence of antibodies to SARS-CoV-2, conducted in LMIC, have been published.

**Added value of this study**

This is the first population-based serological survey for SARS-CoV-2 in Peru's capital city, a megacity in an LMIC, estimating a seroprevalence of 20·8% and a prevalence of 25·2% (serological and molecular tests) after the first peak of cases. We included 3212 participants from 797 households and 241 clusters between June 28th and July 9th, 2020. The seroprevalence was equally distributed across age groups and sex, but it was higher in lower socioeconomic status and overcrowded households. 56% of the seropositive population did not report COVID-19 related symptoms.

**Implications of all the available evidence**

This first estimate of SARS-CoV-2 seroprevalence in Lima shows an intense transmission scenario after the first peak, despite the numerous interventions early established by the government in the first month of the pandemic. More than half of the people with SARS-CoV-2 infection remain asymptomatic, which has important public health implications. The results highlight the importance of considering the existing social inequalities for implementing the response to control transmission, such as lockdowns or isolation of cases, which is a challenge for low- and middle-income countries.

Alt-text: Unlabelled box

## 1 Introduction

COVID-19 is a new human respiratory disease caused by the severe acute respiratory syndrome coronavirus 2 (SARS-CoV-2), which has rapidly spread to the rest of the world since its emergence [1]. From December 30th, 2019, through December 27th, 2020, over 79·2 million COVID-19 cases and 1·7 million deaths have been reported globally [2]. Almost 45% of cases and deaths to date have been reported in the region of the Americas, and the United States of America, Peru, Brazil, Mexico, Colombia, and Argentina account for the highest number of cases in this region [2].

The Peruvian Ministry of Health detected the first COVID-19 case on March 5th, 2020, and, by July 9th, the surveillance system has reported 319 646 confirmed cases of COVID-19 [3]. A confirmed case, according to the Ministry of Health guidelines, was defined as an individual with a positive result for either serology or molecular test for SARS-CoV-2. Transmission apparently began in Lima, Peru's capital city, and from there spread to the rest of the country [4]. Lima has one-third of the country's population and accounted for roughly 50% of the cases and deaths reported [5]. Although Peru implemented strict social distancing measures during the initial phase of the epidemic, these measures were not followed by all citizens due in part to informal employment of almost 70% of the population [6,7]. The COVID-19 pandemic has generated a significant burden of morbidity and mortality in Peru, especially in the elderly, with an estimated delay-adjusted case fatality risk (CFR) of 33·1% (95% CrI: 31·7–34·6%) for men 19·2% (95% CrI: 17·9–20·6%) for women, among people aged 60 to 69 years [8].

Despite surveillance efforts to assess the extent of the pandemic, diagnosed cases only account for a fraction of all cases because of the broad clinical spectrum of this disease, ranging from asymptomatic infection to severe illness [9]. In contrast, population-based serosurveys quantify the proportion of the population that has antibodies against SARS-CoV-2. Such studies also provide approximate estimates of the number of susceptible individuals and the proximity to herd immunity thresholds. Therefore, we conducted a population-based serosurvey to estimate the prevalence of antibodies against SARS-CoV-2 in Lima, Peru, stratified by age, sex, region, socioeconomic status (SES), overcrowding, and symptoms.

## 2 Methods

### 2.1 Study design and sampling

We conducted a population-based cross-sectional survey to measure the seroprevalence of antibodies against SARS-CoV-2 in the Lima metropolitan area, including both the Lima and Callao provinces, between June 28th and July 9th, 2020. The study area has an estimated 10·7 million inhabitants, divided into 50 administrative districts [10]. The study was designed following the World Health Organization (WHO) protocol for COVID-19 population-based seroepidemiological studies [11].

Participants were selected using a two-stage sampling stratified by district (43 in Lima and 7 in Callao provinces), with sample clusters of blocks of approximately 120 households as primary sampling units (PSU) and households as secondary sampling units (SSU). Household members present at the time of the survey were invited to participate in the study, regardless of age or sex. The inference level is reliable to Lima. The precision of other disaggregated estimations will depend on their coefficient of variability or sample size. The supplementary material describes Lima's political division and health administrative areas in detail (p 2).

The target population was people of all ages and sex living in urban areas. We excluded collective residences (e.g. military barracks, police stations, student residences, hospitals, hotels); and also individuals with health conditions where finger prick or nasopharyngeal swab was contraindicated, under the influence of alcohol or drugs, or who did not agree to participate.

### 2.2 Data collection

Sampled households were surveyed by a three-people field team: a health staff to apply the questionnaire, another to collect biological samples for diagnostic tests and the transport vehicle driver. Field supervisors ensured protocol compliance. All field workers were trained in protocol procedures, filling up questionnaires, and sample collection procedures under COVID-19 biosafety guidelines.

Consenting participants completed a questionnaire about socio-demographic, epidemiological, clinical, and laboratory data. Responses from children and people with disabilities received assistance from a responsible adult. Questionnaires were applied either in physical or digital format using an XLSform file [12]. Data was captured using an application based on KoBoToolbox [13], and delivered with a smartphone or tablet using the KoboCollect app [14].

### 2.3 Sample collection

Participants were tested onsite with a point-of-care lateral flow immunochromatographic serological assay, to detect IgM and IgG antibodies against SARS-CoV-2, using 20uL of whole blood from finger-prick samples. Results were reported in 15–20 min. A second, different-model rapid test was performed if the first did not provide an exact negative or positive result. If the rapid serological test was negative, a nasopharyngeal swab was collected for a reverse transcription-polymerase chain reaction (RT-PCR) if they agreed. Nasopharyngeal swabs were collected using a virus sampling kit, which contained one sampling tube with 3·5 ml of viral transport medium and two swabs, from YOCON Biology Technology Company. They were transported to the National Laboratory of Respiratory Viruses at Instituto Nacional de Salud (INS), following the required cold chain. All participants with positive rapid serological or molecular test results were referred for management and follow-up, according to the Peruvian Ministry of Health COVID-19 care protocols.

### 2.4 Laboratory methods

#### 2.4.1 SARS-CoV-2 serology

We used an SD BIOSENSOR, Inc. STANDARD Q COVID-19 IgM/IgG Combo lateral flow point-of-care rapid immunochromatographic assay to detect anti-SARS-CoV-2 antibodies against the nucleocapsid. The manufacturer reported a sensitivity of 96·94% (*N* = 95/98) from RT-PCR confirmed specimens from 14 days after symptoms onset, a specificity of 96·23% (*N* = 255/265), and no cross-reactivity with a set of different pathogens, including other respiratory viruses [15]. Local performance evaluation of the assay was done by INS. Sensitivity was 100% (98·3−100%) among 30 known RT-PCR positive samples and 100% (98·3−100%) diagnostic specificity among 50 RT-PCR negatives. Cross-reactivity was found in two (4%) specimens among 50 pre-COVID-19 (2018–9) plasma and serum samples RT-PCR negative to SARS-CoV-2 and positive to known pathogens. Further details are available in the Supplementary Material (p 2).

#### 2.4.2 Molecular test for SARS-CoV-2

We detected SARS-CoV-2 RNA using a real-time RT-PCR following a previously described standardised protocol [9,16]. Results were reported to participants within 48 hours after sample collection.

### 2.5 Definitions

A seropositive case was defined as an individual with a positive result in a SARS-CoV-2 serology test for either IgG or IgM. According to the definition of a confirmed case in the Peruvian Ministry of Health guidelines, a prevalent case was an individual with a positive result for either serology or molecular test for SARS-CoV-2. Age groups were formed considering the life stages that the Peruvian Ministry of Health recognises in its regulations. Symptomatic cases are those who had anosmia or ageusia or at least two symptoms compatible with COVID-19: fever, cough, sore throat, general malaise, rhinorrhea, headache, muscle pain, or diarrhoea. Oligosymptomatic cases are those with only one symptom but not anosmia or ageusia. Asymptomatic cases are cases without any symptoms. Symptomatic cases were further stratified into those whose symptom onset started before or within the last 14 days prior to the study visit. Socioeconomic status (SES) was provided by Instituto Nacional de Estadistica e Informática (INEI), and its calculation was briefly detailed in the supplementary material (p 2). Overcrowding index is defined as a ratio between the number of people living in a household to the number of rooms in it, excluding bathrooms, kitchen, garage, or corridors. A household was defined as overcrowded when the overcrowding index was three or higher [17]. Regions were: Callao, Northern, Southern, Eastern, and Central Lima, as defined by district boundaries (See Supplementary Material, p 2).

### 2.6 Calculation of socioeconomic status

SES was calculated by multivariate stratification. It consisted of assigning a stratum to each sample cluster in the country's urban area based on information from the 2017 population and housing census using multivariate methods. We used factorial analysis, cluster analysis and discriminant analysis. The factorial analysis evaluated the correlation of the variables according to the indicators defined for the model. In the cluster analysis, we use the K-means method to stratify and identify the optimal stratification model. We used the discriminant analysis to know if the stratification in each unit had been correctly defined. The urban area was divided into five strata: high, medium-high, medium, medium-low and low. Twenty indicators were used for the stratification model, including households with water, drainage and the percentage of people with secondary or higher education (supplementary material, pp 2,3).

### 2.7 Sample size

The minimum sample size required was 2928 participants from 976 households in 244 sample clusters selected among 16,981 sample clusters in Lima (Table S10). A minimum of three participants was expected per household, and four households were selected within each sampled cluster. We used an expected seroprevalence of 10% based on Madrid, Spain, [18] a 1·8 sampling error, a design effect of 1·5 expected to account for household-level clustering, and a non-response rate of 18% due to absences or rejection. Further details in the Supplementary Material (pp 3–54).

### 2.8 Statistical analysis

We estimated the seroprevalence of COVID-19 as the proportion of seropositive individuals in the study sample. As a secondary outcome, the prevalence as the proportion of seropositive or RT-PCR positives. Both seroprevalence estimates were adjusted by sampling weights and test performance and stratified by sex, age groups, SES, region, and categories of overcrowding and symptoms. Among seropositive cases, we estimated the proportion of asymptomatic cases, those with contact with a suspect or confirmed case, and self-reported previous testing. A casewise, complete-data analysis was conducted excluding participants with any missing data. To estimate weighted seroprevalences, we set up the multi-stage sampling using the srvyr R package [19]. Given that we used an imperfect serological assay, we incorporate the test uncertainty [20] using the serosurvey R package [21].

To estimate prevalence ratios, we fit generalised linear regression models to the survey data, with inverse-probability weighting and design-based standard errors. The outcome distribution for seropositivity was defined by a Poisson distribution with log link function. Multivariable regression models were adjusted for sex, age groups, region and socioeconomic status (SES), defined as potential confounders. We used a complete case analysis without imputation. We applied the svyglm function from the survey R package [22]. Further details in Supplementary Material (p 5).

### 2.9 Ethical approval and consent to participate

The Institutional Review Board of INS in Peru reviewed and approved the study protocol on May 27th, 2020 (code: OC-013–20). All participants provided written informed consent before participating in study activities. Individuals under 18 years old were asked to provide written informed assent and consent from a parent or tutor at home.

### 2.10 Role of funding sources

This research was supported by the Peruvian Ministry of Health's regular budget as part of the epidemiological surveillance activities. The funding source did not have any role in the study design, collection, analysis, or interpretation of the data, the writing of this manuscript, or the decision to submit it for publication.

## 3 Results

### 3.1 Sampling and context

**Fig. 1:**
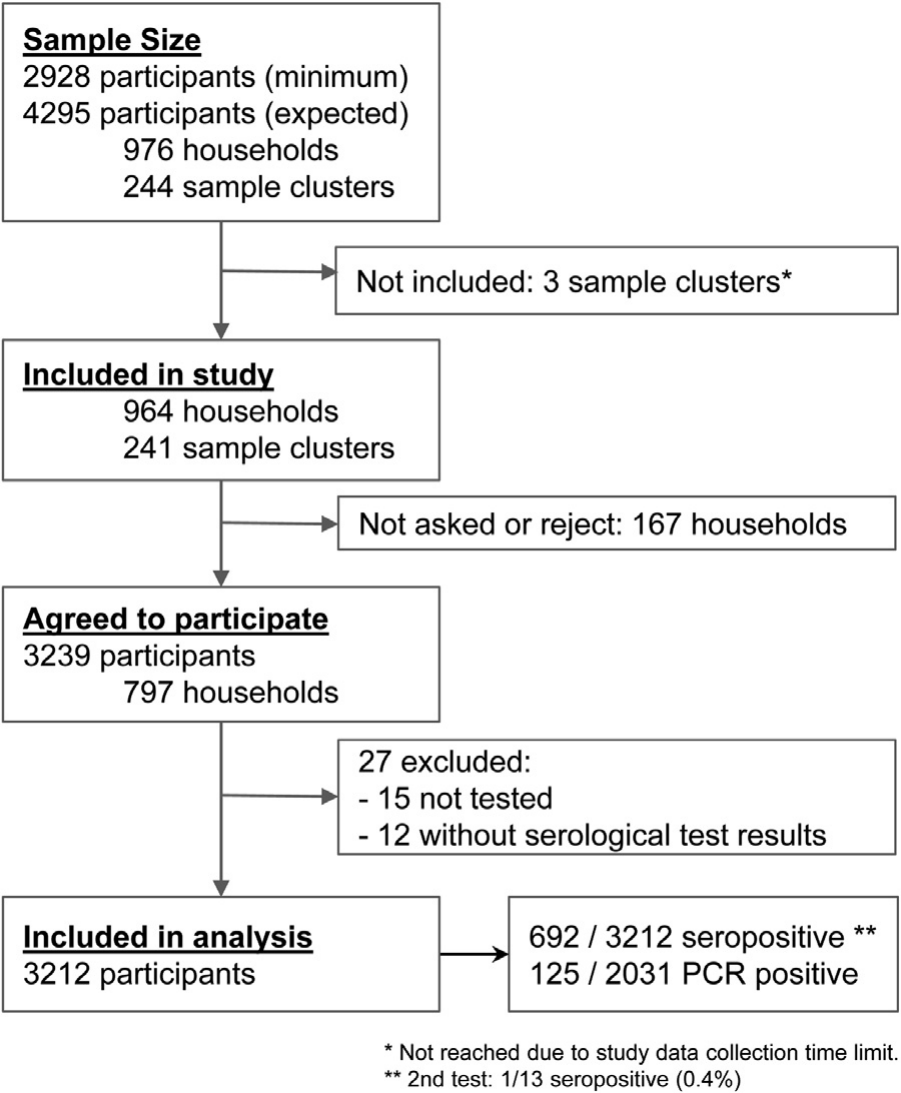
Flowchart of participants from the SARS-CoV-2 seroprevalence study in Lima, Peru: June 28th–July 9th, 2020.

A total of 244 clusters from Lima were randomly selected; however, three of the selected clusters could not be included due to the time limit for data collection; none of the assigned teams could reach them to collect data. Among 964 households in the 241 clusters, 167 households did not participate because they were closed or could not be located (88·6%), or because members refused to participate (11·4%). A total of 797 households and 3239 residents (98·3% of all household residents) agreed to participate, but 15 individuals were not tested, and 12 additional individuals did not have serological results, excluding them from the analysis. Complete data from 3212 participants (99·2%) was analysed (Fig. 1). Among 2520 seronegative participants, 457 rejected the nasopharyngeal swab, and 41 did not obtain RT-PCR results.

Socio-demographic characteristics like sex, province of residence, electricity availability, overcrowding, and type of health insurance of the study sample were comparable to those of the 2017 census population, except in the proportion of children <5 years old (4·1% vs 7·3%, Table S1) and males 25–60 years old (19·5% vs 24·5%, Figs. S1 and S2, Table S2). The serosurvey lasted 12 days and started 115 days after the first case was detected and three days before the end of the national lockdown and the relaxation of stay-at-home orders in some regions of the country. The serosurvey took place in epidemiological weeks (EW) 27–28, after the first peak of the official case and death rates in Lima (EW 22 and 21, respectively) (Fig. S3 and Table S3).

### 3.2 Seroprevalence and associated factors

**Fig. 2:**
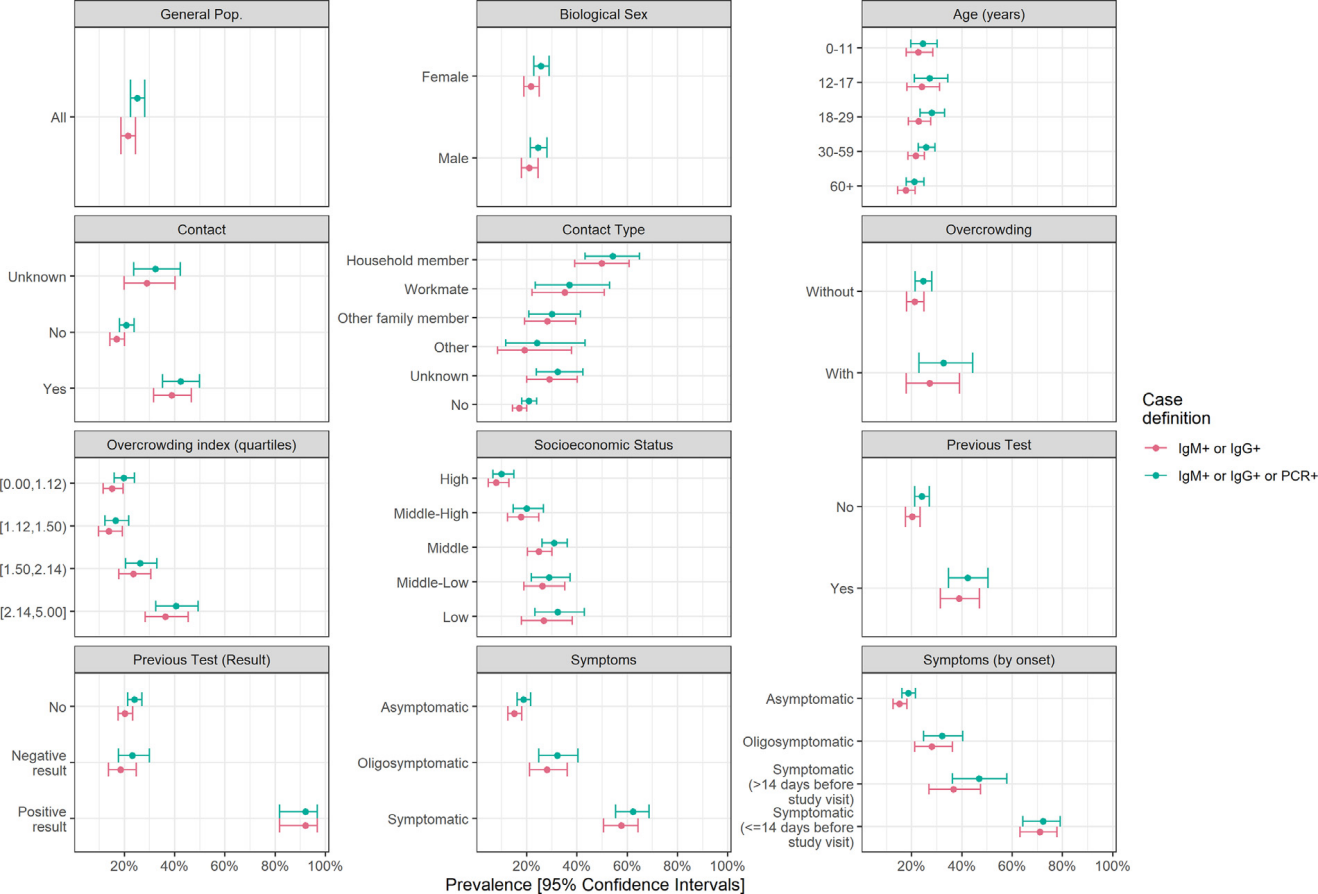
Dot and whisker plot with SARS-CoV-2 prevalence by case definitions across covariates in Lima, Peru: June 28th–July 9th, 2020. Dots represent the sampling weights adjusted point prevalence. Whiskers or error bars represent the 95% Confidence Interval of each point estimate.

The overall SARS-CoV-2 seroprevalence in Lima was 20·8% (95% CI 17·2–23·5), representing approximately 2485,713 (95% CI 2126,908 - 2844,519) people who acquired the infection. The seroprevalence was similar between men and women (19·8% versus 21·4%, aPR=0·96 [95%CI 0·85–1·09, *p* = 0·547]), and across age groups including ≥60 versus ≤11 years old (14·6% versus 21·2%, aPR=0·96 [95%CI 0·73–1·27, *p* = 0·783]) (Tables 1, 3 and Fig. 2). Seroprevalence was highest in Callao but not different to Lima province (27·2% versus 19·6%, aPR=1·37 [95%CI 0·94–2·00, *p* = 0·100], Tables 1, 3 and Fig. S2). Seroprevalence was highest in the lowest SES (25·8%; 95%CI 16·6–36·4), a gradual decrease in SES was associated with a gradual increase in seroprevalence (aPR=2·24 [95%CI 1·27–3·96, *p* = 0·006] in medium-high SES; and aPR=3·41 [95%CI 1·90–6·12, *p*<0·001] in low SES, Tables 1 and 3). About the ethnicity of participants, the point estimate of seroprevalence was higher in mestizos 22·6% (95%CI 18·8–24·5), but not statistically significant concerning the other ethnic groups (Tables 1 and 3).

**Table 1:** Seroprevalence of SARS-CoV-2 by general characteristics.

Characteristics				Participants		Unweighted seroprevalence	Weighted seroprevalence	Weighted seroprevalence adjusted for test uncertainty	%CV *
				Total	n	% (95% CI)	% (95% CI)	% (95% CI)	
Overall				3212	692	21·5 (20·1 - 23·0)	21·4 (18·6 - 24·5)	20·8 (17·2 - 23·5)	6·9
Sex									
	Female			1784	390	21·9 (20·0 - 23·9)	21·8 (18·8 - 25·0)	21·4 (18·3 - 24·1)	7·2
	Male			1428	302	21·1 (19·1 - 23·4)	21·0 (17·8 - 24·5)	19·8 (16·6 - 23·5)	8·1
Age groups (years)									
	0–11			459	102	22·2 (18·5 - 26·3)	22·7 (17·9 - 28·4)	21·2 (16·7 - 27·5)	11·7
	12–17			259	60	23·2 (18·2 - 28·8)	24·0 (18·1 - 31·1)	23·1 (17·0 - 30·1)	13·6
	18–29			570	133	23·3 (19·9 - 27·0)	22·8 (18·6 - 27·6)	20·6 (17·5 - 26·1)	10·0
	30–59			1303	283	21·7 (19·5 - 24·1)	21·6 (18·5 - 25·1)	18·5 (17·6 - 24·5)	7·8
	≥ 60			621	114	18·4 (15·4 - 21·6)	17·6 (14·4 - 21·4)	14·6 (13·4 - 20·2)	10·1
Province									
	Callao			447	125	28·0 (23·8 - 32·4)	28·2 (18·7 - 40·1)	26·9 (16·4 - 39·0)	18·6
	Lima			2765	567	20·5 (19·0 - 22·1)	20·3 (17·6 - 23·5)	18·9 (17·8 - 22·9)	7·3
		Central Lima		873	159	18·2 (15·7 - 20·9)	18·8 (13·8 - 25·0)	17·0 (11·5 - 23·1)	15·0
		Eastern Lima		405	94	23·2 (19·2 - 27·6)	22·7 (15·9 - 31·3)	20·0 (12·1 - 30·3)	16·6
		Northern Lima		725	163	22·5 (19·5 - 25·7)	22·2 (16·4 - 29·3)	21·3 (14·5 - 28·1)	14·3
		Southern Lima		762	151	19·8 (17·0 - 22·8)	19·6 (15·2 - 24·9)	18·0 (14·2 - 23·6)	12·2
Socioeconomic status									
	High			469	38	8·1 (5·8 - 11·0)	7·9 (4·7 - 12·9)	5·2 (3·5 - 11·3)	24·4
	Middle-High			605	109	18·0 (15·0 - 21·3)	17·8 (12·4 - 24·9)	15·4 (10·1 - 23·8)	16·8
	Middle			916	228	24·9 (22·1 - 27·8)	24·8 (20·2 - 30·0)	22·6 (18·5 - 28·3)	9·7
	Middle-Low			822	208	25·3 (22·4 - 28·4)	26·2 (18·9 - 35·2)	25·2 (17·7 - 35·1)	15·3
	Low			400	109	27·3 (22·9 - 31·9)	26·8 (17·8 - 38·2)	25·8 (16·6 - 36·4)	17·2
Overcrowding									
	Without			2121	441	20·8 (19·1 - 22·6)	20·4 (17·0 - 24·3)	20·0 (15·8 - 23·8)	9·1
	With			381	112	25·8 (20·1 - 32·2)	27·2 (17·9 - 39·1)	26·0 (15·9 - 38·2)	15·9
Symptoms compatible with COVID-19 **									
	Asymptomatic			2538	387	15·2 (13·9 - 16·7)	15·1 (12·6 - 18·1)	14·2 (9·8 - 17·1)	9·2
	Oligosymptomatic			254	73	28·7 (23·3 - 34·7)	28·1 (21·2 - 36·2)	26·9 (19·7 - 35·7)	13·5
	Symptomatic			403	232	57·6 (52·6 - 62·4)	57·6 (50·6 - 64·3)	58·6 (50·6 - 64·5)	6·0
		≤14 days before study visit		154	57	37·0 (29·4 - 45·2)	36·6 (27·0 - 47·4)	36·2 (25·8 - 47·1)	14·1
		>14 days before study visit		243	172	70·8 (64·6 - 76·4)	71·0 (63·1 - 77·8)	71·1 (63·4 - 80·2)	5·2
Contact with suspected or confirmed case **									
	No			2172	371	17·1 (15·5 - 18·7)	17·0 (14·3 - 20·0)	14·8 (14·0 - 19·3)	8·5
	Unknown			398	113	28·4 (24·0 - 33·1)	29·0 (20·0 - 40·1)	27·2 (19·2 - 39·5)	17·4
	Yes			524	205	39·1 (34·9 - 43·4)	38·9 (31·7 - 46·6)	38·3 (30·9 - 45·9)	9·7
			Household member	265	132	49·8 (43·6 - 56·0)	49·9 (39·1 - 60·7)	48·9 (38·2 - 62·2)	10·9
			Another family member	141	41	29·1 (21·7 - 37·3)	28·3 (19·1 - 39·6)	27·3 (17·5 - 39·1)	17·8
			Workmate	50	18	36·0 (22·9 - 50·8)	35·1 (22·1 - 50·8)	34·1 (20·7 - 50·5)	19·4
			Other	64	12	18·8 (10·1 - 30·5)	19·2 (8·4 - 37·9)	17·5 (5·7 - 37·1)	34·3
Ethnicity (by self-identification)									
	Mestizo			2796	630	22·5 (21·0 - 24·1)	22·5 (19·5 - 25·9)	22·6 (18·8 - 24·5)	7·2
	Quechua			122	22	18·0 (11·7 - 26·0)	17·1 (10·8 - 25·9)	15·2 (8·9 - 24·5)	20·6
	White			108	15	13·9 (8·0 - 21·9)	12·8 (5·4 - 27·6)	11·0 (3·2 - 26·3)	39·7
	Other, including Afro-descendant			70	8	11·4 (5·1 - 21·3)	11·2 (5·3 - 22·1)	8·7 (3·1 - 20·5)	32·9
		Afro-descendant		29	5	17·2 (5·8 - 35·8)	17·2 (4·4 - 48·4)	***	39·3
		Other		41	3	7·3 (1·5 - 19·9)	7·0 (2·0 - 21·8)	***	49·8

* CV: Coefficient of Variation. ** Self-reported characteristics. *** Not estimated due to lower bound of the confidence interval.

Seroprevalence in overcrowded households was 26·0% (95%CI 15·9–38·2). An increased overcrowding index was associated with higher seroprevalence (aPR=1·41 [95%CI 1·01–1·97, *p* = 0·044] and aPR=1·99 [95%CI 1·41–2·81, *p*<0·001] in the third and fourth quartile, respectively). Seroprevalence was also associated with contact with a suspected or confirmed COVID-19 case, whether a household member (48·9%, aPR=2·67 [95%CI 2·06–3·47, *p*<0·001]), other family members that not reside in the same household (27·3%, aPR=1·66 [95%CI 1·15–2·40, *p* = 0·008]) or a workmate (34·1%, aPR=2·26 [95%CI 1·53–3·35, *p*<0·001]) (Tables 1 and 3). Higher seroprevalence was also observed in participants who reported symptoms. It was even greater among those who said symptoms started 14 days before the serosurvey compared to participants who reported never presenting COVID-19 compatible symptoms (58·6% and 71·1% versus 14·2%, aPR=3·56 [95%CI 2·91–4·37, *p*<0·001] and aPR=4·33 [95%CI 3·52–5·34, *p*<0·001], Tables 1 and 3).

### 3.3 Prevalence and associated factors

RT-PCR molecular assays among agreeing seronegatives added 5·0% positivity beyond the 20·2% seroprevalence estimate, for a SARS-CoV-2 infection prevalence of 25·2% (95% CI 22·5–28·2). The prevalence was slightly higher in 18–29 and 12–17 years old but similar across age groups (28·0% and 27·2%, PR=1·03 [95%CI 0·98–1·09, *p* = 0·213] and PR=1·03 [95%CI 0·96–1·09, *p* = 0·406], not shown in tables). Like seroprevalence, the increased prevalence was observed associated with lower SES, contact with a suspected or confirmed case of COVID-19, and living in overcrowded conditions (Table S5).

### 3.4 Characteristics of seropositive and prevalent cases

The characteristics of seropositive cases revealed important epidemiological features. Most seropositive cases were either asymptomatic (56·1%, Table 2) or reported having had anosmia or at least two COVID-19 compatible symptoms 14 days before the serosurvey (25·4%). Few said having had such symptoms 14 days before the serosurvey (8·2%). Prevalent cases presented the same pattern (Table S6).

**Table 2:** Proportion of self-reported characteristics within SARS-CoV-2 seropositive population.

Characteristics			Seropositive participants	Unweighted prevalence	Weighted prevalence	%CV *
			*N* = 692			
			n	% (95% CI)	% (95% CI)	
Symptoms compatible with COVID-19						
	Asymptomatic		387	55·9 (52·1 - 59·7)	56·1 (49·7 - 62·3)	5·7
	Oligosymptomatic		73	10·5 (8·4 - 13·1)	10·3 (7·8 - 13·5)	13·8
	Symptomatic		232	33·5 (30·0 - 37·2)	33·9 (28·6 - 39·6)	8·2
		≤14 days before study visit	57	8·2 (6·3 - 10·5)	8·2 (5·8 - 11·3)	16·7
		>14 days before study visit	172	24·9 (21·7 - 28·3)	25·4 (20·6 - 30·9)	10·2
Contact with suspected or confirmed case						
	No		371	53·6 (49·8 - 57·4)	54·5 (46·1 - 62·6)	7·7
	Unknown		113	16·3 (13·7 - 19·3)	16·4 (11·5 - 22·9)	17·4
	Yes		205	29·6 (26·2 - 33·2)	29·3 (23·4 - 36·0)	10·9
		Household member	132	19·1 (16·2 - 22·2)	18·9 (13·7 - 25·6)	15·9
		Another family member	41	5·9 (4·3 - 8·0)	5·8 (3·9 - 8·5)	19·6
		Workmate	18	2·6 (1·5 - 4·1)	2·6 (1·6 - 4·1)	23·4
		Other	12	1·7 (0·9 - 3·0)	1·8 (0·9 - 3·6)	36·2
Reported a previous test						
	No		575	83·1 (80·1 - 85·8)	83·2 (78·9 - 86·7)	2·4
	Yes		117	16·9 (14·2 - 19·9)	16·8 (13·3 - 21·1)	11·7
		Negative	40	5·8 (4·2 - 7·8)	5·7 (4·1 - 8·0)	16·7
		Positive	77	11·1 (8·9 - 13·7)	11·1 (8·0 - 15·1)	15·9

* CV: Coefficient of Variation.

**Table 3:** Associated variables to SARS-CoV-2 seropositivity.

Characteristics		Simple models *			Multivariable models *			Model sets **
		PR^	95% CI	p	aPR	95% CI	p	
Socioeconomic status (SES)								A
	High	Ref.						
	Middle-High	2·26	1·27 - 4·04	0·006	2·24	1·27 - 3·96	0·006	
	Middle	3·16	1·88 - 5·29	<0·001	3·13	1·87 - 5·24	<0·001	
	Middle-Low	3·33	1·90 - 5·83	<0·001	3·22	1·86 - 5·59	<0·001	
	Low	3·41	1·90 - 6·09	<0·001	3·41	1·90 - 6·12	<0·001	
Overcrowding Index (by quartiles)								B
	[0·00,1·12)	Ref.			Ref.			
	[1·12,1·50)	0·91	0·61 - 1·36	0·654	0·86	0·58 - 1·25	0·427	
	[1·50,2·14)	1·56	1·09 - 2·23	0·015	1·41	1·01 - 1·97	0·044	
	[2·14,5·00]	2·40	1·71 - 3·38	<0·001	1·99	1·41 - 2·81	<0·001	
Symptoms compatible with COVID-19								C
	Asymptomatic	Ref.			Ref.			
	Oligosymptomatic	1·86	1·41 - 2·45	<0·001	1·79	1·36 - 2·35	<0·001	
	Symptomatic ***	3·81	3·14 - 4·62	<0·001	3·56	2·91 - 4·37	<0·001	
	≤14 days before study visit	2·42	1·81 - 3·24	<0·001	2·34	1·72 - 3·20	<0·001	
	>14 days before study visit	4·70	3·85 - 5·72	<0·001	4·33	3·52 - 5·34	<0·001	
Contact with suspected or confirmed case								D
	No	Ref.			Ref.			
	Unknown	1·71	1·18 - 2·48	0·005	1·74	1·2 - 2·53	0·004	
	Yes ***	2·29	1·78 - 2·94	<0·001	2·17	1·71 - 2·74	<0·001	
	Household member	2·94	2·24 - 3·86	<0·001	2·67	2·06 - 3·47	<0·001	
	Another family member	1·66	1·14 - 2·43	0·009	1·66	1·15 - 2·40	0·008	
	Workmate	2·07	1·36 - 3·15	<0·001	2·26	1·53 - 3·35	<0·001	
	Other	1·13	0·57 - 2·22	0·727	1·01	0·50 - 2·04	0·968	
Ethnicity (by self-identification)								E
	Mestizo	Ref.			Ref.			
	Quechua	0·76	0·50 - 1·16	0·199	0·74	0·49 - 1·11	0·148	
	White	0·57	0·26 - 1·25	0·160	0·70	0·34 - 1·43	0·327	
	Other, including Afro-descendant ***	0·50	0·26 - 0·96	0·039	0·52	0·27 - 1.00	0·051	
	Afro-descendant	0·76	0·35 - 1·67	0·496	0·71	0·32 - 1·60	0·412	
	Other	0·31	0·12 - 0·83	0·021	0·35	0·13 - 0·97	0·046	

* Survey-weighted generalised linear regression models. ** A: multiple model adjusted by sex, age groups, province. From B to E: each multiple model adjusted by sex, age groups, regions and SES. ^ Prevalence Ratio. *** Used first, then it was disaggregated as in rows below.

Most seropositive cases reported no previous contact with suspect or confirmed cases (54·5%, 95%CI 46·1–62·6) and 16·4% (95%CI 11·5–22·9) said they did not know if they had contact. Identified contacts were household members (18·9%), other family members (5·8%), or workmates (2·6%). Finally, only 16·8% (95%CI 13·3–21·1) of all seropositive cases reported having had a previous test, and 11·1% (95%CI 8·0–15·1) reported having had a previous positive test.

## 4 Discussion

This is the first population-based serological survey for SARS-CoV-2 in Lima, estimating a seroprevalence of 20·8%, and a prevalence of 25·2% after the first peak. This was equally distributed across age, sex, and regions. Seroprevalence was higher in lower SES, in overcrowded households, and in subjects with suspected or confirmed contact within the household or workmates. 56% of the seropositive population did not report COVID-19 related symptoms, and almost two-thirds did not report contact with suspect or confirmed COVID-19 cases.

The high estimated prevalence rate shows intense transmission despite the widespread quarantine established by the government in the first months of the pandemic. Lima is a megacity with more than 10 million inhabitants, with a high population density (3697 people per square kilometer) and high informal employment (58·4%) [6,23]. Informal workers have precarious income, little access to social protection and health care. They have to continue working and mobilising daily to earn a living, from suburbs with precarious and very crowded public transportation, facilitating transmission. These factors could have limited the population's capability to follow strict quarantine and social distancing measures; they could have contributed to the high transmission observed, with higher seroprevalence in lower SES and overcrowded households [7,24]. One study found that cities with spatial factors, such as higher crowding and population density, could have longer epidemics and higher attack rates after the first epidemic wave, even after the implementation of lockdowns [25].

Lima seroprevalence is one of the highest among reported capital cities in Latin America. As far as we know, in Latin America, only Brazil, Chile, and Ecuador had published a population-based, a school, and a rural community report, respectively [26,27]. Lima had a higher estimate than the most populated cities in Brazil, including Rio de Janeiro (7·5%) and São Paulo (2·3%) [28]. Outside Latin America, the closest estimate to Lima was Delhi in India (23%) [29]. Furthermore, the seroprevalence was higher than London in England (13%) [30] and Madrid in Spain (11·3%), [18] where the capital city had higher estimates compared to the rest of the country.

Being the capital city, Lima had a lower seroprevalence than those reported from other cities in Peru, contrary to the observed regional distribution of estimates from England [30] and Spain [18]. For instance, Lambayeque in the northern coast (29·8%), [31] Cusco in the southern Andes (38·1%) [32], and Iquitos in the Amazon Basin (71%), [33] although these studies are not strictly comparable in methodological and analytical procedures since some differences were observed: in the inclusion criteria participants were older than 9 or 18 years, some excluded health workers, only one participant per household was enrolled, and in another, the seroprevalence was not adjusted according to the test's sensitivity and specificity. Cusco and Iquitos present socioeconomic differences regarding Lima, with informal employment rates greater than 80% and higher monetary poverty levels and overcrowding [6,34]. This pattern is also observed in Brazil, where mostly all the Northern Region had a prevalence rate higher than 10%, including cities like Belem (17%) and Boa Vista (25%) [28], Manaus (66%) [35] and Maranhao (40%) [36]. In Brazil, this pattern was associated with SES [28].

People under 12 years old (21·2%) had a seroprevalence as high as the rest of the age groups, showing that the proportion of susceptibles in Lima is almost equal across ages. These estimates highly contrast with the cumulative rate of cases until EW 28 captured by the surveillance system, underestimating younger ages (Fig. S3). This prevalence in children is in opposition to a current review that concluded that children are the age group with the lowest seroprevalence and risk based on population-based studies from European countries and two U.S. states [27]. Additionally, the observed seroprevalence in children under 12 from Lima was higher than elementary grade students between 6 and 12 (10·8%) from a school community in Chile [37]. In contrast, the lowest seroprevalence observed in older adults over 60 could be a consequence of survival bias due to their high mortality rate (Fig. S3); 69% of deaths belong to people older than 60 years old [3,5]. The proportion of the population susceptible to being infected by SARS-CoV-2 is still high in all age groups. This implies that if physical distancing interventions are relaxed, possibly by opening schools or allowing gatherings, older adults could once again become the most affected group due to their high vulnerability, considering that they may live in multi-generational households that include children [38].

Low SES and overcrowded households were associated with higher seroprevalence, similar to the findings in Brazil. However, in this study, crowded conditions were measured considering only the household size (living with six or more people) [28]. We observed a gradient between the household overcrowding index and seroprevalence. Overcrowded households are related to low income or underemployment [39] and have been commonly associated with high rates of acute respiratory infection like tuberculosis in Latin America [40]. Also, seroprevalence was higher in subjects with self-reported contact with a suspected or confirmed case within the household (49·2%) and cases among other family members who did not reside in the same household (27·3%). Similar conclusions were observed in Spain (31%), [18] Pakistan (52% secondary attack rate) [41], and England (9%) [30]. Our results also reveal a high prevalence of infection in those who reported contact with confirmed cases in the workplace (34%) compared to Spain, where a prevalence of 10·6% was observed, [18] but we did not obtain more data about the working conditions for further discussion. These suggest that overcrowding and family gatherings could have played an essential role in the transmission of SARS-CoV-2, despite the quarantine and restrictions about gatherings that the government established. These make visible the challenges of applying physical distance interventions and lockdowns in cities of high population density, high informal employment, and economic inequalities [42]. Also, the data reinforce the importance of early detection and isolation of cases in centres of temporary isolation and not at households.

Almost two-thirds of the seropositive population did not report COVID-19 related symptoms or were oligosymptomatic. Lima showed higher asymptomatics compared to previous reviews (40%−45%), [43] cruise ship (46·5%) [44], and population-based studies (32%) [30]. Compared to Spain, [18] Peru had more asymptomatic cases (56% vs 33%) but lower oligosymptomatic ones (10% vs 49%). These differences could be affected by recall bias due to its self-report collection four months after the start of the SARS-CoV-2 epidemic, in contrast to one or two months in those studies, thus overestimating the asymptomatic proportion. The estimated proportion of asymptomatic not detected by the epidemiological surveillance system may lay between 1,124,583 and 1,652,697 people, a large population that could have played a significant role in the transmission.

Equally important, high rates of denied or unknown contact with either suspect or confirmed cases (70%) among the seropositive population could be attributed to some factors, such as a limited implementation of an active contract tracing strategy. This was also observed in the low proportion of previous tests within seropositive (17%), a high rate of asymptomatic cases, and low adherence to self-protection measurements. Therefore, it is crucial to implement an effective contact tracing strategy, starting from the early detection and isolation of cases, to prevent spread and identify the most common infection sources, as done in other Latin American settings [45]. It is essential to recognize that this strategy's implementation requires intensive labour and resources allocation, which represents a significant challenge for the response of health systems in low and middle-income countries, given the rapid increase in the incidence of cases.

Our study's key strengths are its representative sample, the usage of standardised protocols, and the application of molecular tests. First, the random two-stage sampling and a sample size above the minimum required allowed an optimal representation of the urban population. This resulted in estimates with low coefficients of variability when stratified by age, sex, and other disaggregated estimations. Second, we based the study design on WHO protocols for population-based seroepidemiological studies to increase its comparability across countries [11]. We complemented it with an open data acquisition tool and analysis workflow to allow its reproducibility, review, and improvement. Third, this serological survey included molecular tests for seronegative participants. This allowed us to additionally estimate the prevalence, including the active infections without detectable antibodies. Finally, our results were similar to a study carried out in Brazil [28], revealing the critical association between SES and overcrowding with SARS-CoV-2 transmission.

These results should be interpreted considering certain limitations. First, our sample slightly underrepresented the men adult population. This could be caused by low adherence to lockdown due to increased economic insecurity during the pandemic, possibly underestimating seroprevalence. Second, we could not register the survey's non-response rate due to an inconsistent registry in the forms during fieldwork. However, the study design, a household coverage of 82%, covered residents in households of 98%, a sample size larger than the minimum expected, and a sample that was representative when comparing the demographic and economic characteristics with the expected population, support the representativeness and validity of study findings. Third, the local test performance study suffers from spectrum bias due to hospitalised patients' primary enrollment to define sensitivity and bias it upwards, potentially underestimating the true seroprevalence [46]. Even though the main advantage of these performance results was the inclusion of local circulating pathogens assessing the test's cross-reactivity, in contrast to the manufacturer. Fourth, household clustering of positive cases could aggregate correlated participants and overestimate seroprevalence. Though this was considered in the sample size calculation with a design effect factor, as in previous studies, [18] and the internal correlation structure of sampling units is already handled by considering the cluster sampling.

In conclusion, our study is the first estimate of the seroprevalence of SARS-CoV-2 in Lima, the capital city of Peru, which showed a high prevalence after the first peak of cases. That shows an intense transmission scenario in a megacity despite the lockdowns, restrictions on the mobilization of the population, and closure of borders, early established by the government in the first month of the pandemic. The equal proportion of susceptibles across age groups and regions shows that the most vulnerable groups still need to be protected, and physical distancing interventions must not be relaxed. The study was carried out with a reliable and standardised methodology, obtaining results that show widespread transmission in the population of Lima, with a higher seroprevalence in lower SES, in overcrowded households, and in subjects who had contact with cases within the home, in addition to a significant proportion of cases asymptomatic and those who denied or were unaware of having had contact with cases. This highlights the importance of considering the existing social inequalities in this megacity for implementing the response to control transmission, such as lockdowns, isolation of cases, and contact tracing, which is a challenge for low- and middle-income countries.

## Data sharing statement

Anonymised data that support the findings of this study are available upon request following institutional requirements to this email: cdc.investigacion@dge.gob.pe. The survey questionnaire as an XLSform and analysis code are available in a GitHub repository and archived in Zenodo [47].

## Funding

This research was supported by the regular budget of Centro Nacional de Epidemiología, Prevención y Control de Enfermedades (CDC Peru) and INS from the Peruvian Ministry of Health as part of the epidemiological surveillance activities. Dirección de Redes Integradas de Salud (DIRIS) of Lima and Dirección Regional de Salud (DIRESA) Callao also collaborated with their resources from regular activities of the rapid response teams and epidemiological surveillance.

## Disclaimer

The views expressed in this article are those of the authors. They do not necessarily reflect the official policy or position of the CDC Peru, Peruvian Ministry of Health, nor the Peruvian Government.

## Author contributions

M.R. and C.M. conceptualised and designed the study; M.R., C.M., J.V. and M.F. developed the statistical design; M.R., F.C., M.V., C.M., G.S. and K.M. were executive coordinators and led the relationship with regional health services and logistics; F.C. was responsible for choosing the serological tests, laboratory protocols and test performance study; F.C. and K.M. developed the operational protocols for fieldwork; M.R., F.C., G.S., K.M., and CDC working group members were responsible for training the involved administrative and health personnel. INS working group members performed the laboratory analyses; M.R., C.M., G.S., K.M. and A.V.developed the statistical analysis plan, table and figure design; M.R., G.S., K.M. and A.V. had direct access to the raw data; M.R., G.S., K.M. and A.V. retrieved, managed data, and performed quality control evaluations; J.V. and M.F. calculated the sampling weights; A.V. was in charge of the statistical analyses; M.R., G.S., K.M. and A.V. interpreted the results and wrote the first draft of the manuscript. F.C., M.V., C.M., J.V. and M.E. critically reviewed the first draft. All authors and working members approved the final version and agreed to be accountable for the work.

## Declaration of Competing Interest

Dr. Margot Vidal declared having been Executive Director of the Directorate of Noncommunicable Diseases of the National Center for Public Health at INS during the study's execution. The other authors have no conflicts of interest.

## Appendix Supplementary materials

Image, application 1

Image, application 2
