# Supplementary material for: SARS-CoV-2 prevalence associated to low socioeconomic status and overcrowding in an LMIC megacity: A population-based seroepidemiological survey in Lima, Peru
Source: eClinicalMedicine. 2021 Mar 30;34:100801. doi: 10.1016/j.eclinm.2021.100801 (PMC8009628; doi:10.1016/j.eclinm.2021.100801)
Supplement: Supplementary file 1 [file mmc1.docx]

**SUPPLEMENTARY MATERIAL**

Contents

Peru COVID-19 Working Group

Supplementary Methods

Supplementary Table 1

Supplementary Table 2

Supplementary Table 3

Supplementary Table 4

Supplementary Table 5

Supplementary Table 6

Supplementary Table 7

Supplementary Table 8

Supplementary Table 9

Supplementary Table 10

Supplementary Table 11

Supplementary Figure 1

Supplementary Figure 2

Supplementary Figure 3

Questionnaire

References
