# Supplementary material for: SARS-CoV-2 prevalence associated to low socioeconomic status and overcrowding in an LMIC megacity: A population-based seroepidemiological survey in Lima, Peru
Source: eClinicalMedicine. 2021 Mar 30;34:100801. doi: 10.1016/j.eclinm.2021.100801 (PMC8009628; doi:10.1016/j.eclinm.2021.100801)
Supplement: Supplementary file 2 [file mmc2.docx]

**SUPPLEMENTARY MATERIAL**

**PERU COVID-19 WORKING GROUP**

Centro Nacional de Epidemiología, Prevención y Control de Enfermedades, Peruvian Ministry of Health

- Dante Castro Garro
- Isabel Sandoval Ordinola
- Lenin Yonel La Torre Rosillo
- Idania Edith Mamani Pilco
- Javier Masías Mimbela
- Mario Neyser Vásquez Domínguez
- Karina Vargas Quispe
- David Enriquez Cusi
- Jesús Chuquihuaccha Cabrera
- Martha Calderón Silva
- Elizabeth Guevara Cachay
- Natalia Borgoño Espinoza
- Iván Vargas Meneses
- Armando Llamocca
- Fabiola Gil Cipirán
- Noemí Flores Jaime

Instituto Nacional de Salud, Peruvian Ministry of Health

- Maribel Carmen Huaringa Núñez
- Priscila Nayu Lope Pari
- Johanna Nery Balbuena Torres
- Ana Jorge Berrocal

**SUPPLEMENTARY METHODS**

**Additional data sources**

We used socioeconomic summarized data at the province administrative area level from the 2017 National Population Census´s publicly available results.[^1^](https://paperpile.com/c/pdhLeo/KrGjN) We used population estimates by age and sex for the year 2020 from the Ministry of Health Open Repository.[^2^](https://paperpile.com/c/pdhLeo/8C8Uo) We imported intervention data by governments from the UNESCO COVID-19 Impact on Education Dataset[^3^](https://paperpile.com/c/pdhLeo/60vuH) using the covid19viz R package.[^4^](https://paperpile.com/c/pdhLeo/z1Bug) We imported COVID-19 daily reports of cases[^5^](https://paperpile.com/c/pdhLeo/4ANsH) and deaths[^6,7^](https://paperpile.com/c/pdhLeo/qHVj6+VYGJl) from National Open Data repositories using the covidPeru R package.[^8^](https://paperpile.com/c/pdhLeo/olKXX)

**Political division and Health administrative areas of Lima**

Lima Metropolitan Area is formed by the province of Lima and Callao.[^9^](https://paperpile.com/c/pdhLeo/gNgmY) This is spatially divided by five health administrative areas.[^2^](https://paperpile.com/c/pdhLeo/8C8Uo) These areas are officially named Dirección de Redes Integradas de Salud (DIRIS) within the province of Lima or Dirección Regional de Salud (DIRESA) for Callao Region. The province of Lima is divided in four health administrative areas each composed of a set of districts: i) Northern Lima (Ancon, Carabayllo, Comas, Independencia, Los Olivos, Puente Piedra, Rímac, San Martin De Porres, Santa Rosa), ii) Central Lima (Lima, Breña, Jesus Maria, La Victoria, Lince, Magdalena Del Mar, Pueblo Libre, Miraflores, San Borja, San Isidro, San Juan De Lurigancho, San Luis, San Miguel, Surquillo), iii) Eastern Lima (Ate, Chaclacayo, Cieneguilla, El Agustino, La Molina, Lurigancho, Santa Maria De Huachipa, Santa Anita), and iv) Southern Lima (Barranco, Chorrillos, Lurín, Pachacamac, Pucusana, Punta Hermosa, Punta Negra, San Bartolo, San Juan De Miraflores, Santa Maria Del Mar, Santiago De Surco, Villa El Salvador, Villa Maria Del Triunfo).

**Local performance evaluation of SARS-CoV-2 Point-Of-Care Serology Test**

A local performance evaluation of the assay was done by the Peruvian National Institute of Health (INS). It used 30 COVID-19 positive samples mostly from hospitalized patients (95%) with more than seven days after symptoms onset and 50 COVID-19 negative samples from the currently healthy population after the introduction of SARS-CoV-2. Sensitivity was 100% (98·3%-100%) among known RT-PCR positive samples and 100% (98·3%-100%) diagnostic specificity among RT-PCR negatives.

Cross-reactivity was assessed in 50 pre-COVID-19 (2018-9) plasma and serum samples RT-PCR negative to SARS-CoV-2 but positive to known pathogens (*Treponema pallidum*, Leptospira, Chikungunya, Dengue, Zika, HIV, HTLV, Hepatitis, Cytomegalovirus, and syphilis among pregnant women), five samples per pathogen. Two specimens (4%) were positive, one out of five with Chikungunya IgM+ and one out of five with Zika IgM+. Specimens positive to other respiratory viruses were not available, given that diagnostic samples are usually nasopharyngeal swabs instead of blood. No performance test reports were available in public repositories for this specific product.[^10,11^](https://paperpile.com/c/pdhLeo/T6Cz1+uVukz)

The second rapid serology test was performed with a different label (whole/serum/Plasma. Zhejiang Orient Gene, Biotech Co LTD, China).[^12^](https://paperpile.com/c/pdhLeo/GFZQa) A local performance test evaluation is available elsewhere.[^13^](https://paperpile.com/c/pdhLeo/HLgrM) This was used in 13 participants only (Figure 1).

**Calculation of socioeconomic status**

The indicators used for the stratification model were:

- Percentage of households with refrigerators

- Percentage of households with a television

- Percentage of households with a radius

- Percentage of households with a computer

- Percentage of households with internet

- Percentage of households with cable

- Percentage of households with a washing machine.

- Percentage of households with a telephone.

- Percentage of households with a different floor of land.

- Percentage of households with a television, refrigerator, computer and telephone.

- Percentage of households with a television, refrigerator, radio, and washing machine.

- Percentage of households with a telephone, internet and computer.

- Percentage of households with transportation

- Percentage of households with small domestic appliances

- Percentage of households with water

- Percentage of households with drainage

- Percentage of people with ESSALUD health insurance

- Percentage of people with university education

- Percentage of people with secondary education or higher

- Percentage of women with economic activity 14 years and older.

**Sample size calculation**

For this estimate, we used an expected seroprevalence of 10% based on the observed seroprevalence of Madrid, Spain[^14^](https://paperpile.com/c/pdhLeo/qi5vx) and a 1·8 sampling error. A design effect of 1·5 was expected to account for household-level clustering, and the estimated non-response rate due to absences or rejection was set to 18%. We used population estimates from the 2017 National Population Census.[^15^](https://paperpile.com/c/pdhLeo/JGaiS) We expected to achieve adequate statistical precision with a coefficient of variability of 6·1%. The distribution of the number of sample clusters selected per district was proportional to the estimated official cumulative incidence of confirmed COVID-19 cases by April 14th, 2020.

To determine the sample size for the survey, the statistical reliability requirements for the study´s proposed scope have been taken into account. A 95% confidence level is considered for the calculation. The following expression was used:

$$n=\frac{N \times P \times Q \times Z^{2} \times deff^{2} \times TNR}{[(N-1)\times d^{2} + (Z^{2} \times P\times Q)]}$$

Where:

$n$: size of the sample of people.

$N$: Number of persons in the study area (N=10000000)

$P$: is the estimated prevalence of SARS-CoV-2 for the study setting. (P=0·10). To determine the use of 0·10 we used the estimate obtained in the seroprevalence study of Spain, using a probabilistic sample: national prevalence of 5% with a prevalence of 10% in Madrid, the capital city.[^14^](https://paperpile.com/c/pdhLeo/qi5vx)

$$Q = 1- P$$

$Z = 1\cdot96$ (for 95% confidence)

$TNR = 18\%$ : expected non-response rate due to the absence or rejection of informants.

$deff = 1\cdot5$ : design effect due to sample clustering.

$d = 0\cdot018$ : precision level (sampling error)

As can be seen in Table S10, 2928 individuals were estimated as the minimum sample size. However, from the number of selected households and given that the average number of people per household in Lima and Callao is 4, according to the 2017 National Population Census, and the number of homes per household is 1·1, on average, the expected sample size is 4295 individuals.

The number of households to be selected is 976.

It is proposed to select 4 households for each sample cluster in the sample, so the number of sample clusters to be selected is equal to 244.

It is expected that the estimate of the prevalence of SARS-CoV-2 in the study area will have a statistical precision (CV) of 6·1%, which is considered good.

**Sample Distribution**

Considering the magnitude (number of confirmed cases) of SARS-CoV-2 by district, the Table S11 presents the distribution of sample clusters by district.

**Sampling weights calculation**

To make the survey estimates representative of the population, it is necessary to multiply the data for each household in the database by the sample weight or expansion factor.

Each record’s final expansion factor has two components: the basic sampling factor and the non-response adjustment factors.

The basic sampling factor for each sampled household is determined by the sample design, which is the inverse of its final probability of selection.

The sample design of the ESPI-SARS-CoV-2 Lima study involves two stages of sampling, the units were selected with probabilities proportional to the size of the household in the first stage, and the second stage a systematic sampling with random start.

The probability of selection of each household will be calculated through the following formula:

$$p_{i}=\frac{g * M_{i}}{M}x\frac{m_{i}}{M_{i}}$$

Where:

$p_{i}$: Final selection probability of the ith Primary Sampling Unit (PSU) selected.

$g$: Sample of PSU to be selected in Lima.

$M$: Total number of households in the framework Lima.

$M_{i}$: Total number of households in the framework for the i-th PSU selected in Lima.

$m_{i}$: Sample of selected households in the i-th PSU selected

The basic expansion factor is calculated as the inverse of the final selection probability:

$$w_{i}=\frac{1}{p_{i}}$$

It is important to adjust the expansion factors taking into account the magnitude of non-response. Since the expansion factors are calculated at each selected PSU level, it is advantageous to adjust the expansion factors at this level.

In this case, the final expansion factors (${w'}_{i}$) for the selected households in the i-th PSU selected can be expressed as:

$$w'_{i}=w_{i}*\frac{m_{i}}{m'_{i}}$$

Where:

$m_{i}$: Sample of selected homes in the i-th selected PSU.

${m'}_{i}$: Sample of households surveyed in the ith selected PSU.

**Statistical analysis**

We used sampling weights to adjust the seroprevalence estimates and take into account the multistage sampling uncertainty. We calculated sampling weights per sample cluster after the data collection using the final selection probability and the observed response at each sample cluster. Further details in the Supplementary Material. Sampling weights were included in a two-stage cluster sample set up by sample cluster and household, stratified by districts. We estimated prevalence 95% confidence intervals using the "logit" method. This method uses the srvyr R package to fit a logistic regression model, computes Wald-type log-odds scale intervals, and then transforms them to prevalence estimates.[^16^](https://paperpile.com/c/pdhLeo/VShmi)

Given that we used an imperfect test, we incorporate the test uncertainty into the sampling design uncertainty. We applied a hierarchical Bayesian method to estimate the posterior probability of seroprevalence for a test with uncertain performance.[^17^](https://paperpile.com/c/pdhLeo/QIB6k) As inputs, we used the expanded point estimate number of positive tests and the total population that resulted from the sampling weights adjusted seroprevalence estimation. To maintain the sampling design uncertainty, we applied the same method for each confidence interval. We used the reported number of true positives, false positives, true negatives, and false negatives from the local test performance evaluation. Seroprevalence estimates were computed and expressed as posterior medians based on 10000 samples from the Bayesian posterior distribution. We implemented this workflow in the serosurvey R package.[^18^](https://paperpile.com/c/pdhLeo/SsrlK)

To estimate prevalence ratios, we fit generalized linear regression models to the survey data, with inverse-probability weighting and design-based standard errors. The outcome distribution was defined by a Poisson distribution with log link function. Multivariable regression models were adjusted for sex, age groups, region and socioeconomic status (SES). We used a complete case analysis without imputation. We applied the svyglm function from the survey R package[^19^](https://paperpile.com/c/pdhLeo/sufYe) and the analysis workflow from the epitidy R package.[^20^](https://paperpile.com/c/pdhLeo/grU86)

**SUPPLEMENTARY TABLES**

**Table S1.** Characteristics from the eligible population and selected sample.

**Table S2.** Age and sex percent difference between the selected sample and estimated population in Lima, Peru 2020.

**Table S3.** Number of COVID-19 confirmed cases and deaths from surveillance systems in Lima, Peru.

**Table S4.** Government intervention dates due to COVID-19 pandemic, Peru 2020.

**Table S5.** Prevalence of SARS-CoV-2 by general characteristics.

**Table S6.** Proportion of self-reported characteristics within SARS-CoV-2 seropositive and RT-PCR positive population.

**Table S7.** Seroprevalence of SARS-CoV-2 by age groups and region.

**Table S8.** Characteristics of subjects included in the survey analysis.

**Table S9.** Self-reported characteristics of subjects included in the survey analysis.

**Table S10.** Estimated sample sizes and the expected variability or precision indicator (CV).

**Table S11.** Distribution of sample clusters by district.

| **Table S1. Characteristics from eligible population and selected sample.** | | | | |
| --- | --- | --- | --- | --- |
| Characteristics | | Population ^ | Sample | N * |
|  |  | 2017 | 2020 |  |
|  |  | N = 9569468 | N=3212 |  |
|  |  | N (%) | N (%) |  |
| Sex: | |  |  | 3212 |
|  | Female | 4915123 (52·2) | 1784 (55·5) |  |
|  | Male | 4654345 (49·4) | 1428 (44·5) |  |
| Age (year): | |  |  | 3212 |
|  | 0-4 | 696911 ( 7·3) | 133 (4·1) |  |
|  | 5-9 | 729615 ( 7·6) | 218 (6·8) |  |
|  | 10-14 | 714153 ( 7·5) | 229 (7·1) |  |
|  | 15-29 | 2447544 (25·6) | 708 (22·0) |  |
|  | 30-44 | 2216258 (23·2) | 730 (22·7) |  |
|  | 45-64 | 1552849 (16·2) | 715 (22·3) |  |
|  | 65+ | 1212138 (12·7) | 479 (14·9) |  |
| Province: | |  |  | 3212 |
|  | Callao | 975968 (10·4) | 447 (13·9) |  |
|  | Lima | 8436399 (89·6) | 2765 (86·1) |  |
| Electricity: ** | |  |  |  |
|  | No | 90949 ( 3·1) | 58 (2·2) |  |
|  | Yes | 2798269 (96·98) | 2526 (97·8) |  |
| Overcrowding: | |  |  | 2334 |
|  | No | 8619190 (91·6) | 2121 (90·9) |  |
|  | Yes | 793177 ( 8·4) | 213 (9·1) |  |
| Health insurance: | |  |  | 3195 |
|  | SIS | 2648106 (27·7) | 975 (30·5) |  |
|  | None | 2578389 (26·9) | 781 (24·4) |  |
|  | Other | 4342973 (45·4) | 1439 (45·0) |  |
| RT-PCR: | |  |  | 2031 |
|  | Negative |  | 1906 (93·8) |  |
|  | Positive |  | 125 (6·2) |  |
| Serology: | |  |  | 3212 |
|  | Seronegative |  | 2520 (78·5) |  |
|  | Seropositive |  | 692 (21·5) |  |
| ^ Census Population from the 2017 National Census. * N: Complete number of observations from collected sample. All population proportions were calculated within the Census Population, except: ** Among number of private dwellings, and *** Among population in private dwellings with occupants present. Following details available in the Geo Peru data dictionary. | | | | |

| **Table S2. Age and sex percent difference between selected sample and estimated population in Lima, Peru 2020.** | | | | | | |
| --- | --- | --- | --- | --- | --- | --- |
| Sex | Age | Population ^ | | Sample | | Difference |
|  | (years) | N | **%** | **N** | % | **%** |
| Male | <0 | 67189 | 0·63 | 4 | 0·12 | -0·50 |
|  | 01-04 | 305603 | 2·84 | 67 | 2·09 | -0·76 |
|  | 05-09 | 321172 | 2·99 | 112 | 3·49 | 0·50 |
|  | 10-14 | 343248 | 3·20 | 123 | 3·83 | 0·63 |
|  | 15-19 | 325783 | 3·03 | 118 | 3·67 | 0·64 |
|  | 20-24 | 387170 | 3·60 | 114 | 3·55 | -0·05 |
|  | 25-29 | 422114 | 3·93 | 85 | 2·65 | -1·28 |
|  | 30-34 | 419124 | 3·90 | 118 | 3·67 | -0·23 |
|  | 35-39 | 418397 | 3·89 | 101 | 3·14 | -0·75 |
|  | 40-44 | 403549 | 3·76 | 82 | 2·55 | -1·20 |
|  | 45-49 | 350136 | 3·26 | 86 | 2·68 | -0·58 |
|  | 50-54 | 329376 | 3·07 | 79 | 2·46 | -0·61 |
|  | 55-59 | 285242 | 2·66 | 75 | 2·33 | -0·32 |
|  | 60-64 | 216930 | 2·02 | 61 | 1·90 | -0·12 |
|  | 65-69 | 190791 | 1·78 | 64 | 1·99 | 0·22 |
|  | 70-74 | 151681 | 1·41 | 43 | 1·34 | -0·07 |
|  | 75-79 | 102476 | 0·95 | 48 | 1·49 | 0·54 |
|  | 80+ | 123781 | 1·15 | 48 | 1·49 | 0·34 |
| Female | <0 | 65131 | 0·61 | 4 | 0·12 | -0·48 |
|  | 01-04 | 292484 | 2·72 | 58 | 1·81 | -0·92 |
|  | 05-09 | 326432 | 3·04 | 106 | 3·30 | 0·26 |
|  | 10-14 | 352156 | 3·28 | 106 | 3·30 | 0·02 |
|  | 15-19 | 364752 | 3·40 | 105 | 3·27 | -0·13 |
|  | 20-24 | 486397 | 4·53 | 147 | 4·58 | 0·05 |
|  | 25-29 | 473287 | 4·41 | 139 | 4·33 | -0·08 |
|  | 30-34 | 426677 | 3·97 | 129 | 4·02 | 0·04 |
|  | 35-39 | 442347 | 4·12 | 147 | 4·58 | 0·46 |
|  | 40-44 | 423380 | 3·94 | 153 | 4·76 | 0·82 |
|  | 45-49 | 396529 | 3·69 | 130 | 4·05 | 0·36 |
|  | 50-54 | 352136 | 3·28 | 110 | 3·42 | 0·15 |
|  | 55-59 | 300281 | 2·80 | 93 | 2·90 | 0·10 |
|  | 60-64 | 248252 | 2·31 | 81 | 2·52 | 0·21 |
|  | 65-69 | 212926 | 1·98 | 99 | 3·08 | 1·10 |
|  | 70-74 | 152900 | 1·42 | 65 | 2·02 | 0·60 |
|  | 75-79 | 112808 | 1·05 | 43 | 1·34 | 0·29 |
|  | 80+ | 149922 | 1·40 | 69 | 2·15 | 0·75 |
| ^ Population estimates from the 2020 REUNIS data repository. | | | | | | |

| **Table S3.** Incident and cumulative COVID-19 confirmed cases and deaths from surveillance systems in Lima, Peru.* | | | | | | | | | | | | | |
| --- | --- | --- | --- | --- | --- | --- | --- | --- | --- | --- | --- | --- | --- |
| Surveillance source | EW first day | EW | Type | 0-11 years | | 12-17 years | | 18-29 years | | 30-59 years | | 60+ years | |
|  |  |  |  | N | Rate | N | Rate | N | Rate | N | Rate | N | Rate |
| Cases | 2020-03-01 | 10 | Incident | 1 | 0·06 | 0 | 0 | 2 | 0·1 | 1 | 0·02 | 1 | 0·06 |
|  |  |  | Cumulative | 1 | 0·06 | 0 | 0 | 2 | 0·1 | 1 | 0·02 | 1 | 0·06 |
|  | 2020-04-05 | 15 | Incident | 152 | 9·23 | 84 | 10·54 | 993 | 47·52 | 2854 | 62·76 | 824 | 49·56 |
|  |  |  | Cumulative | 191 | 11·6 | 106 | 13·3 | 1417 | 67·81 | 4056 | 89·2 | 1236 | 74·35 |
|  | 2020-05-10 | 20 | Incident | 328 | 19·93 | 235 | 29·48 | 3300 | 157·92 | 10163 | 223·5 | 2485 | 149·48 |
|  |  |  | Cumulative | 1790 | 108·75 | 1020 | 127·94 | 12671 | 606·37 | 39513 | 868·96 | 11713 | 704·56 |
|  | 2020-06-14 | 25 | Incident | 294 | 17·86 | 192 | 24·08 | 1849 | 88·48 | 6632 | 145·85 | 2427 | 145·99 |
|  |  |  | Cumulative | 3626 | 220·29 | 2196 | 275·45 | 28261 | 1352·43 | 91190 | 2005·42 | 27318 | 1643·22 |
|  | 2020-07-19 | 30 | Incident | 294 | 17·86 | 239 | 29·98 | 2188 | 104·71 | 7519 | 165·36 | 2598 | 156·27 |
|  |  |  | Cumulative | 4805 | 291·91 | 3177 | 398·5 | 36843 | 1763·12 | 122502 | 2694·02 | 38473 | 2314·21 |
| Deaths | 2020-03-01 | 10 | Incident | 0 | 0 | 0 | 0 | 0 | 0 | 0 | 0 | 0 | 0 |
|  |  |  | Cumulative | 0 | 0 | 0 | 0 | 0 | 0 | 0 | 0 | 0 | 0 |
|  | 2020-04-05 | 15 | Incident | 1 | 0·06 | 0 | 0 | 0 | 0 | 59 | 1·3 | 113 | 6·8 |
|  |  |  | Cumulative | 1 | 0·06 | 0 | 0 | 3 | 0·14 | 92 | 2·02 | 199 | 11·97 |
|  | 2020-05-10 | 20 | Incident | 2 | 0·12 | 0 | 0 | 3 | 0·14 | 244 | 5·37 | 577 | 34·71 |
|  |  |  | Cumulative | 10 | 0·61 | 2 | 0·25 | 25 | 1·2 | 1027 | 22·59 | 2318 | 139·43 |
|  | 2020-06-14 | 25 | Incident | 0 | 0 | 0 | 0 | 4 | 0·19 | 202 | 4·44 | 516 | 31·04 |
|  |  |  | Cumulative | 15 | 0·91 | 5 | 0·63 | 60 | 2·87 | 2131 | 46·86 | 4940 | 297·15 |
|  | 2020-07-19 | 30 | Incident | 0 | 0 | 2 | 0·25 | 1 | 0·05 | 168 | 3·69 | 373 | 22·44 |
|  |  |  | Cumulative | 23 | 1·4 | 9 | 1·13 | 74 | 3·54 | 2971 | 65·34 | 6952 | 418·17 |
| * Between March 1st - August 1st, 2020. Population estimates by age group from the 2020 REUNIS data repository. Rates per 100K habitants. EW: Epidemiological Week. | | | | | | | | | | | | | |

| **Table S4. Government intervention dates due to COVID-19 pandemic, Peru 2020.** | | |
| --- | --- | --- |
| **Date start** | **Date finish** | **Intervention** |
| 2020-03-16 | 2020-06-30 | Closed due to COVID-19 |
| 2020-06-28 | 2020-07-09 | Seroprevalence study |
| 2020-07-01 | 2020-08-01 | Partially open |

| **Table S5. Prevalence of SARS-CoV-2 by general characteristics.** | | | | | | | | |
| --- | --- | --- | --- | --- | --- | --- | --- | --- |
| Characteristics | | | | Participants | | Unweighted prevalence | Weighted prevalence | %CV * |
|  |  |  |  | Total | n | % (95% CI) | % (95% CI) |  |
| Overall | | | | 3212 | 817 | 25·4 (23·9 - 27·0) | 25·2 (22·5 - 28·2) | 5·7 |
| Sex | | | |  |  |  |  |  |
|  | Female | | | 1784 | 462 | 25·9 (23·9 - 28·0) | 25·7 (22·8 - 28·9) | 6·0 |
|  | Male | | | 1428 | 355 | 24·9 (22·6 - 27·2) | 24·6 (21·4 - 28·1) | 6·9 |
| Age in groups (years) | | | |  |  |  |  |  |
|  | 0-11 | | | 459 | 111 | 24·2 (20·3 - 28·4) | 24·5 (19·7 - 30·2) | 10·8 |
|  | 12-17 | | | 259 | 69 | 26·6 (21·4 - 32·5) | 27·2 (21·1 - 34·4) | 12·3 |
|  | 18-29 | | | 570 | 162 | 28·4 (24·8 - 32·3) | 28·0 (23·4 - 33·1) | 8·8 |
|  | 30-59 | | | 1303 | 339 | 26·0 (23·7 - 28·5) | 25·8 (22·6 - 29·3) | 6·6 |
|  | ≥ 60 | | | 621 | 136 | 21·9 (18·7 - 25·4) | 21·1 (17·8 - 24·9) | 8·6 |
| Socioeconomic status | | | |  |  |  |  |  |
|  | High | | | 469 | 49 | 10·4 ( 7·8 - 13·6) | 10·0 ( 6·6 - 14·9) | 19·7 |
|  | Middle-High | | | 605 | 122 | 20·2 (17·0 - 23·6) | 20·0 (14·6 - 26·7) | 14·6 |
|  | Middle | | | 916 | 284 | 31·0 (28·0 - 34·1) | 30·9 (26·1 - 36·1) | 8·0 |
|  | Middle-Low | | | 822 | 230 | 28·0 (24·9 - 31·2) | 28·9 (21·8 - 37·3) | 13·2 |
|  | Low | | | 400 | 132 | 33·0 (28·4 - 37·8) | 32·3 (23·2 - 43·0) | 13·9 |
| Province | | | |  |  |  |  |  |
|  | Lima | | | 2765 | 685 | 24·8 (23·2 - 26·4) | 24·5 (21·8 - 27·5) | 6·0 |
|  | Callao | | | 447 | 132 | 29·5 (25·3 - 34·0) | 29·6 (20·3 - 41·1) | 17·2 |
| Overcrowding | | | |  |  |  |  |  |
|  | Without | | | 2121 | 508 | 24·0 (22·1 - 25·8) | 23·6 (20·3 - 27·3) | 7·6 |
|  | With | | | 213 | 67 | 31·5 (25·3 - 38·2) | 32·8 (22·9 - 44·4) | 15·8 |
| Symptoms compatible with COVID-19 ** | | | |  |  |  |  |  |
|  | Asymptomatic | | | 2538 | 482 | 19·0 (17·5 - 20·6) | 18·8 (16·2 - 21·6) | 7·3 |
|  | Oligosymptomatic | | | 254 | 83 | 32·7 (26·9 - 38·8) | 32·1 (24·8 - 40·4) | 12·3 |
|  | Symptomatic | | | 403 | 251 | 62·3 (57·4 - 67·0) | 62·3 (55·4 - 68·8) | 5·4 |
|  |  | ≤14 days before study visit | | 154 | 73 | 47·4 (39·3 - 55·6) | 46·9 (36·3 - 57·8) | 11·6 |
|  |  | >14 days before study visit | | 243 | 175 | 72·0 (65·9 - 77·6) | 72·3 (64·2 - 79·2) | 5·2 |
| Contact with suspected or confirmed case ** | | | |  |  |  |  |  |
|  | No | | | 2172 | 457 | 21·0 (19·3 - 22·8) | 20·8 (18·0 - 23·9) | 7·2 |
|  | Unknown | | | 398 | 127 | 31·9 (27·4 - 36·7) | 32·4 (23·8 - 42·3) | 14·4 |
|  | Yes | | | 524 | 222 | 42·4 (38·1 - 46·7) | 42·4 (35·1 - 49·9) | 8·9 |
|  |  | | Household member | 265 | 143 | 54·0 (47·8 - 60·1) | 54·3 (43·3 - 64·9) | 10·0 |
|  |  | | Another family member | 141 | 43 | 30·5 (23·0 - 38·8) | 30·1 (20·8 - 41·4) | 16·8 |
|  |  | | Workmate | 50 | 19 | 38·0 (24·7 - 52·8) | 37·0 (23·4 - 53·0) | 19·1 |
|  |  | | Other | 64 | 15 | 23·4 (13·8 - 35·7) | 24·1 (11·7 - 43·2) | 29·7 |
| Ethnicity (by self-identification) | | | |  |  |  |  |  |
|  | Mestizo | | | 2796 | 736 | 26·3 (24·7 - 28·0) | 26·3 (23·3 - 29·5) | 6·0 |
|  | Quechua | | | 122 | 25 | 20·5 (13·7 - 28·7) | 19·6 (13·3 - 27·9) | 17·6 |
|  | White | | | 108 | 25 | 23·1 (15·6 - 32·2) | 21·8 (10·1 - 40·8) | 33·8 |
|  | Other, including Afro-descendant | | | 70 | 11 | 15·7 ( 8·1 - 26·4) | 15·4 ( 7·6 - 28·7) | 30·9 |
|  |  | | Afro-descendant | 29 | 6 | 20·7 ( 8·0 - 39·7) | 20·5 ( 4·0 - 61·2) | 45·3 |
|  |  | | Other | 41 | 5 | 12·2 ( 4·1 - 26·2) | 11·8 ( 4·7 - 26·5) | 35·9 |
| * CV: Coefficient of Variation. ** Self-reported characteristics. | | | | | | | | |

| **Table S6. Proportion of self-reported characteristics within SARS-CoV-2 seropositive and RT-PCR positive population.** | | | | | | |
| --- | --- | --- | --- | --- | --- | --- |
| Characteristics | | | Positive participants | Unweighted prevalence | Weighted prevalence | %CV * |
|  |  |  | N=817 |  |  |  |
|  |  |  | n | % (95% CI) | % (95% CI) |  |
| Symptoms compatible with COVID-19 | | |  |  |  |  |
|  | Asymptomatic | | 482 | 59·0 (55·5 - 62·4) | 59·1 (53·4 - 64·6) | 4·8 |
|  | Oligosymptomatic | | 83 | 10·2 ( 8·2 - 12·4) | 10·0 ( 7·7 - 13·0) | 13·4 |
|  | Symptomatic | | 251 | 30·7 (27·6 - 34·0) | 31·1 (26·5 - 36·2) | 7·9 |
|  |  | ≤14 days before study visit | 73 | 8·9 ( 7·1 - 11·1) | 8·9 ( 6·6 - 11·9) | 14·9 |
|  |  | >14 days before study visit | 175 | 21·4 (18·7 - 24·4) | 22·0 (17·9 - 26·8) | 10·2 |
| Contact with suspected or confirmed case | | |  |  |  |  |
|  | No | | 457 | 55·9 (52·5 - 59·4) | 57·2 (49·3 - 64·7) | 6·8 |
|  | Unknown | | 127 | 15·5 (13·1 - 18·2) | 15·7 (11·2 - 21·6) | 16·7 |
|  | Yes | | 222 | 27·2 (24·1 - 30·4) | 27·3 (21·8 - 33·6) | 10·9 |
|  |  | Household member | 143 | 17·5 (15·0 - 20·3) | 17·6 (12·8 - 23·7) | 15·6 |
|  |  | Another family member | 43 | 5·3 ( 3·8 - 7·0) | 5·3 ( 3·6 - 7·7) | 19·4 |
|  |  | Workmate | 19 | 2·3 ( 1·4 - 3·6) | 2·3 ( 1·4 - 3·7) | 24·0 |
|  |  | Other | 15 | 1·8 ( 1·0 - 3·0) | 1·9 ( 1·0 - 3·5) | 31·2 |
| Reported a previous test | | |  |  |  |  |
|  | No | | 683 | 83·6 (80·9 - 86·1) | 84·4 (80·6 - 87·5) | 2·1 |
|  | Yes | | 127 | 15·5 (13·1 - 18·2) | 15·6 (12·5 - 19·4) | 11·2 |
|  |  | Negative | 50 | 6·1 ( 4·6 - 8·0) | 6·1 ( 4·6 - 8·2) | 14·9 |
|  |  | Positive | 77 | 9·4 ( 7·5 - 11·6) | 9·5 ( 6·9 - 13·0) | 16·1 |
| * CV: Coefficient of Variation | | | | | | |

| **Table S7. Seroprevalence of SARS-CoV-2 by age groups and region.** | | | | | | | |
| --- | --- | --- | --- | --- | --- | --- | --- |
| Characteristics | | Participants | | Unweighted seroprevalence | Weighted seroprevalence | Weighted seroprevalence adjusted for test uncertainty | %CV * |
|  |  | Total | n | % (95% CI) | % (95% CI) | % (95% CI) |  |
| Age (years) | |  |  |  |  |  |  |
|  | [0,10) | 351 | 76 | 21·7 (17·5 - 26·3) | 22·2 (17·0 - 28·3) | 20·3 (15·6 - 27·1) | 12·9 |
|  | [10,20) | 452 | 104 | 23·0 (19·2 - 27·2) | 23·7 (18·8 - 29·5) | 22·0 (14·9 - 27·8) | 11·4 |
|  | [20,30) | 485 | 115 | 23·7 (20·0 - 27·8) | 23·0 (18·4 - 28·4) | 22·1 (17·0 - 26·2) | 11·0 |
|  | [30,40) | 495 | 109 | 22·0 (18·4 - 25·9) | 21·7 (17·6 - 26·4) | 19·4 (16·4 - 25·6) | 10·2 |
|  | [40,50) | 451 | 104 | 23·1 (19·2 - 27·2) | 23·6 (19·0 - 28·9) | 22·5 (16·9 - 27·7) | 10·6 |
|  | [50,60) | 357 | 70 | 19·6 (15·6 - 24·1) | 19·1 (14·9 - 24·2) | 17·9 (13·5 - 23·0) | 12·3 |
|  | [60,70) | 305 | 61 | 20·0 (15·7 - 24·9) | 19·1 (14·6 - 24·6) | 17·5 (13·3 - 23·7) | 13·2 |
|  | [70,80) | 199 | 41 | 20·6 (15·2 - 26·9) | 20·1 (15·0 - 26·3) | 17·7 (12·8 - 25·1) | 14·1 |
|  | [80,100) | 117 | 12 | 10·3 ( 5·4 - 17·2) | 9·7 ( 5·3 - 17·1) | 7·8 ( 3·3 - 15·3) | 29·0 |
| * CV: Coefficient of Variation. ** Self-reported characteristics. | | | | | | | |

| **Table S8. Characteristics of subjects included in the survey analysis.** | | | | | | |
| --- | --- | --- | --- | --- | --- | --- |
| Characteristics | | ·· | Rapid Serological test | | p* | N** |
|  |  | All | Negative | Positive |  |  |
|  |  | N=3212 | N=2520 | N=692 |  |  |
|  |  | N (%) | N (%) | N (%) |  |  |
| Overall | | 3212 (100·0) | 2520 (78·5) | 692 (21·5) |  | 3212 |
| Sex | |  |  |  | 0·656 | 3212 |
|  | Female | 1784 (55·5) | 1394 (78·1) | 390 (21·9) |  |  |
|  | Male | 1428 (44·5) | 1126 (78·9) | 302 (21·1) |  |  |
| Age in groups (years) | |  |  |  | 0·252 | 3212 |
|  | 0-11 | 459 (14·3) | 357 (77·8) | 102 (22·2) |  |  |
|  | 12-17 | 259 (8·1) | 199 (76·8) | 60 (23·2) |  |  |
|  | 18-29 | 570 (17·7) | 437 (76·7) | 133 (23·3) |  |  |
|  | 30-59 | 1303 (40·6) | 1020 (78·3) | 283 (21·7) |  |  |
|  | ≥ 60 | 621 (19·3) | 507 (81·6) | 114 (18·4) |  |  |
| Socioeconomic status | |  |  |  | <0.001 | 3212 |
|  | High | 469 (14.6%) | 431 (91.9%) | 38 (8.1%) |  |  |
|  | Middle-High | 605 (18.8%) | 496 (82.0%) | 109 (18.0%) |  |  |
|  | Middle | 916 (28.5%) | 688 (75.1%) | 228 (24.9%) |  |  |
|  | Middle-Low | 822 (25.6%) | 614 (74.7%) | 208 (25.3%) |  |  |
|  | Low | 400 (12.5%) | 291 (72.8%) | 109 (27.3%) |  |  |
| Region | |  |  |  | 0·001 | 3212 |
|  | Central Lima | 873 (27·2) | 714 (81·8) | 159 (18·2) |  |  |
|  | Callao Region | 447 (13·9) | 322 (72·0) | 125 (28·0) |  |  |
|  | Eastern Lima | 405 (12·6) | 311 (76·8) | 94 (23·2) |  |  |
|  | Northern Lima | 725 (22·6) | 562 (77·5) | 163 (22·5) |  |  |
|  | Southern Lima | 762 (23·7) | 611 (80·2) | 151 (19·8) |  |  |
| Province | |  |  |  | <0·001 | 3212 |
|  | Lima | 2765 (86·1%) | 2198 (79·5%) | 567 (20·5%) |  |  |
|  | Callao | 447 (13·9%) | 322 (72·0%) | 125 (28·0%) |  |  |
| Overcrowding Index | | 1·4 [1·0;2·0] | 1·3 [1·0;2·0] | 1·8 [1·2;2·3] | <0·001 | 2581 |
| Overcrowding Index by quartiles | |  |  |  | <0·001 | 2581 |
|  | [0·00,1·12) | 819 (31·7) | 691 (84·4) | 128 (15·6) |  |  |
|  | [1·12,1·50) | 473 (18·3) | 406 (85·8) | 67 (14·2) |  |  |
|  | [1·50,2·14) | 766 (29·7) | 584 (76·2) | 182 (23·8) |  |  |
|  | [2·14,5·00] | 523 (20·3) | 336 (64·2) | 187 (35·8) |  |  |
| Overcrowding | |  |  |  | 0·105 | 2334 |
|  | Without | 2121 (90·9) | 1680 (79·2) | 441 (20·8) |  |  |
|  | With | 213 (9·1) | 158 (74·2) | 55 (25·8) |  |  |
| Ethnicity (by self-identification) | |  |  |  | 0·024 | 3096 |
|  | Mestizo | 2796 (90·3) | 2166 (77·5) | 630 (22·5) |  |  |
|  | Quechua | 122 (3·9) | 100 (82·0) | 22 (18·0) |  |  |
|  | White | 108 (3·5) | 93 (86·1) | 15 (13·9) |  |  |
|  | Afro-descendant | 29 (0·9) | 24 (82·8) | 5 (17·2) |  |  |
|  | Other | 41 (1·3) | 38 (92·7) | 3 (7·3) |  |  |
| * p-value for Chi-square, Fisher exact or Mann Whitney test when appropriate. ** Complete number of observations from collected sample. | | | | | | |

| **Table S9. Self-reported characteristics of subjects included in the survey analysis.** | | | | | | | |
| --- | --- | --- | --- | --- | --- | --- | --- |
| Characteristics | | | ·· | Rapid Serological test | | p* | N** |
|  |  |  | All | Negative | Positive |  |  |
|  |  |  | N=3212 | N=2520 | N=692 |  |  |
|  |  |  | N (%) | N (%) | N (%) |  |  |
| Symptoms compatible with COVID-19 | | |  |  |  | <0·001 | 3195 |
|  | Asymptomatic | | 2538 (79·4) | 2151 (84·8) | 387 (15·2) |  |  |
|  | Oligosymptomatic | | 254 (7·9) | 181 (71·3) | 73 (28·7) |  |  |
|  | Symptomatic | | 403 (12·6) | 171 (42·4) | 232 (57·6) |  |  |
|  |  | ≤14 days before study visit | 154 (4·8) | 97 (63·0) | 57 (37·0) |  |  |
|  |  | >14 days before study visit | 243 (7·6) | 71 (29·2) | 172 (70·8) |  |  |
| Contact with suspected or confirmed case | | |  |  |  | <0·001 | 3094 |
|  | No | | 2172 (70·2) | 1801 (82·9) | 371 (17·1) |  |  |
|  | Unknown | | 398 (12·9) | 285 (71·6) | 113 (28·4) |  |  |
|  | Yes | | 524 (16·9) | 319 (60·9) | 205 (39·1) |  |  |
| Contact with suspected or confirmed case | | |  |  |  | <0·001 | 3090 |
|  | No | | 2172 (70·3) | 1801 (82·9) | 371 (17·1) |  |  |
|  | Unknown | | 398 (12·9) | 285 (71·6) | 113 (28·4) |  |  |
|  | Yes | Household member | 265 (8·6) | 133 (50·2) | 132 (49·8) |  |  |
|  |  | Another family member | 141 (4·6) | 100 (70·9) | 41 (29·1) |  |  |
|  |  | Workmate | 50 (1·6) | 32 (64·0) | 18 (36·0) |  |  |
|  |  | Other | 64 (2·1) | 52 (81·2) | 12 (18·8) |  |  |
| Reported a previous test | | |  |  |  | <0·001 | 3120 |
|  | No |  | 2824 (90·5) | 2249 (79·6) | 575 (20·4) |  |  |
|  | Yes |  | 296 (9·5) | 179 (60·5) | 117 (39·5) |  |  |
| Reported a previous test by type | | |  |  |  | <0·001 | 3120 |
|  | No |  | 2824 (90·5) | 2249 (79·6) | 575 (20·4) |  |  |
|  | Yes | PCR | 22 (0·7) | 9 (40·9) | 13 (59·1) |  |  |
|  |  | Point-of-care serological test | 274 (8·8) | 170 (62·0) | 104 (38·0) |  |  |
| Reported a previous test by result | | |  |  |  | <0·001 | 3119 |
|  | No |  | 2824 (90·5) | 2249 (79·6) | 575 (20·4) |  |  |
|  | Yes | Negative | 211 (6·8) | 171 (81·0) | 40 (19·0) |  |  |
|  |  | Positive | 84 (2·7) | 7 (8·3) | 77 (91·7) |  |  |
| Recent work (in the last 14 days) | | |  |  |  | 0·689 | 2892 |
|  | No |  | 2206 (76·3) | 1732 (78·5) | 474 (21·5) |  |  |
|  | Yes |  | 686 (23·7) | 533 (77·7) | 153 (22·3) |  |  |
| Work Area (in the last 14 days) | | |  |  |  | 0·441 | 660 |
|  | Trade | | 221 (33·5) | 171 (77·4) | 50 (22·6) |  |  |
|  | Caregiver | | 15 (2·3) | 13 (86·7) | 2 (13·3) |  |  |
|  | Cleaning | | 25 (3·8) | 18 (72·0) | 7 (28·0) |  |  |
|  | Health | | 43 (6·5) | 36 (83·7) | 7 (16·3) |  |  |
|  | Security | | 20 (3·0) | 12 (60·0) | 8 (40·0) |  |  |
|  | Transport | | 73 (11·1) | 56 (76·7) | 17 (23·3) |  |  |
|  | Others | | 263 (39·8) | 208 (79·1) | 55 (20·9) |  |  |
| * p-value for Chi-square, Fisher exact or Mann Whitney test when appropriate. ** Complete number of observations from collected sample. | | | | | | | |

| **Table S10. Estimated sample sizes and the expected variability or precision indicator (CV)** | | | | |
| --- | --- | --- | --- | --- |
| **Study area** | **Minimum Sample Size** | **Household sample** | **Sample clusters** | **Precision**  **CV%** |
| Total | 2928 | 976 | 244 | 0·061 |

| **Table S11. Distribution of sample clusters by district** | | |
| --- | --- | --- |
| **District** | **Covid-19 confirmed cases until April 13th, 2020** | **Distribution sample clusters per district** |
| Callao | 784 | 15 |
| Lima Cercado | 505 | 10 |
| San Juan de Lurigancho | 505 | 10 |
| San Martín de Porres | 442 | 10 |
| Villa El Salvador | 368 | 9 |
| San Juan de Miraflores | 344 | 8 |
| Santiago de Surco | 327 | 8 |
| Comas | 323 | 8 |
| Villa María del Triunfo | 321 | 8 |
| Chorrillos | 296 | 8 |
| Ate | 281 | 8 |
| El Agustino | 260 | 7 |
| Jesús María | 259 | 7 |
| La Victoria | 227 | 7 |
| Miraflores | 205 | 7 |
| Rímac | 199 | 6 |
| Santa Anita | 173 | 6 |
| Los Olivos | 168 | 6 |
| La Molina | 150 | 6 |
| San Borja | 137 | 5 |
| Independencia | 133 | 5 |
| San Miguel | 132 | 5 |
| San Isidro | 126 | 5 |
| Surquillo | 126 | 5 |
| Carabayllo | 116 | 5 |
| Puente Piedra | 112 | 5 |
| Bellavista | 103 | 5 |
| Breña | 100 | 5 |
| Lince | 82 | 4 |
| Pueblo Libre | 81 | 4 |
| Ventanilla | 78 | 4 |
| Chaclacayo | 59 | 3 |
| Magdalena del Mar | 57 | 3 |
| Barranco | 53 | 3 |
| San Luis | 49 | 3 |
| Lurigancho | 48 | 3 |
| Lurín | 38 | 3 |
| La Perla | 32 | 3 |
| Pachacamac | 26 | 2 |
| Ancón | 22 | 2 |
| C. de la Legua y R. | 22 | 2 |
| Mi Perú | 13 | 2 |
| Pucusana | 7 | 1 |
| Cieneguilla | 6 | 1 |
| Punta Hermosa | 2 | 1 |
| Other districts | 4 | 1 |

**SUPPLEMENTARY FIGURES**

**Figure S1.** Age and sex distribution of study participants against the estimated population in Lima, 2020 (Source: REUNIS)

**Figure S2.** Spatial distribution of SARS-CoV-2 seroprevalence in Lima, Peru

**Figure S3.** Dates of the Seroprevalence study, Government interventions, and Epidemiological surveillance counts of confirmed cases and total deaths during the COVID-19 pandemic in Lima, Peru: March 1st - August 1st, 2020. (Source: UNESCO and Plataforma Nacional de Datos Abiertos Perú).


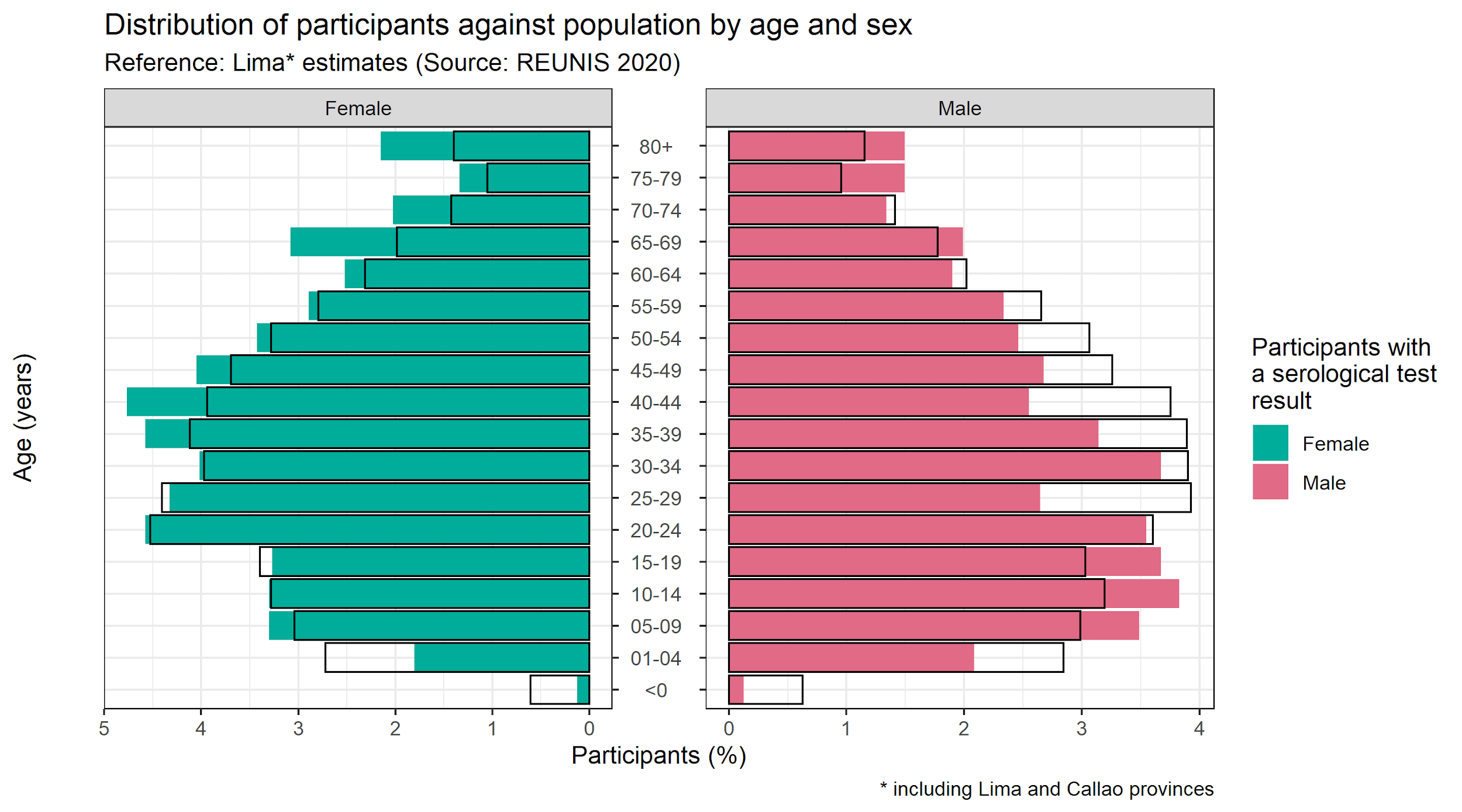


**Figure S1**


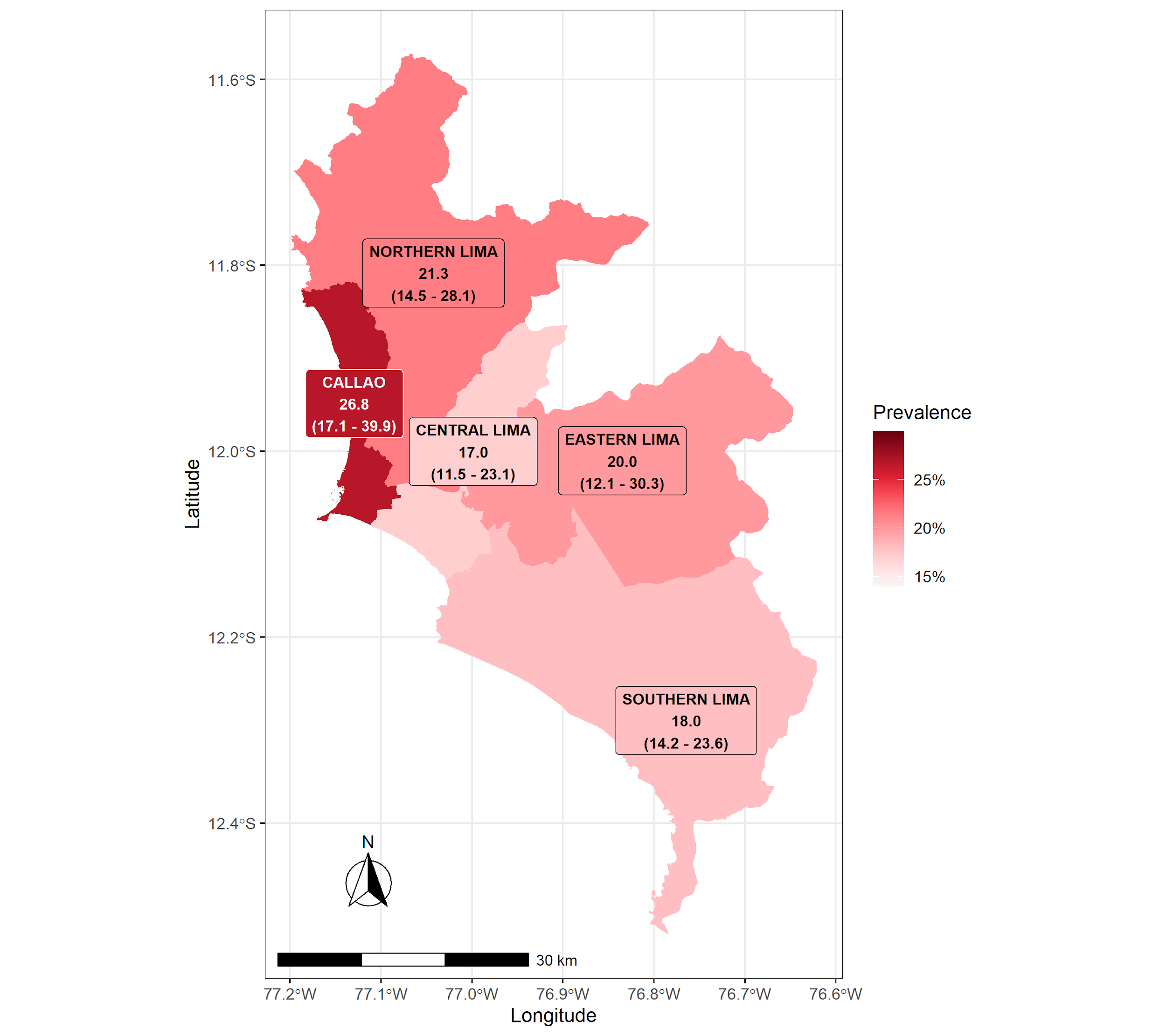


**Figure S2**

**
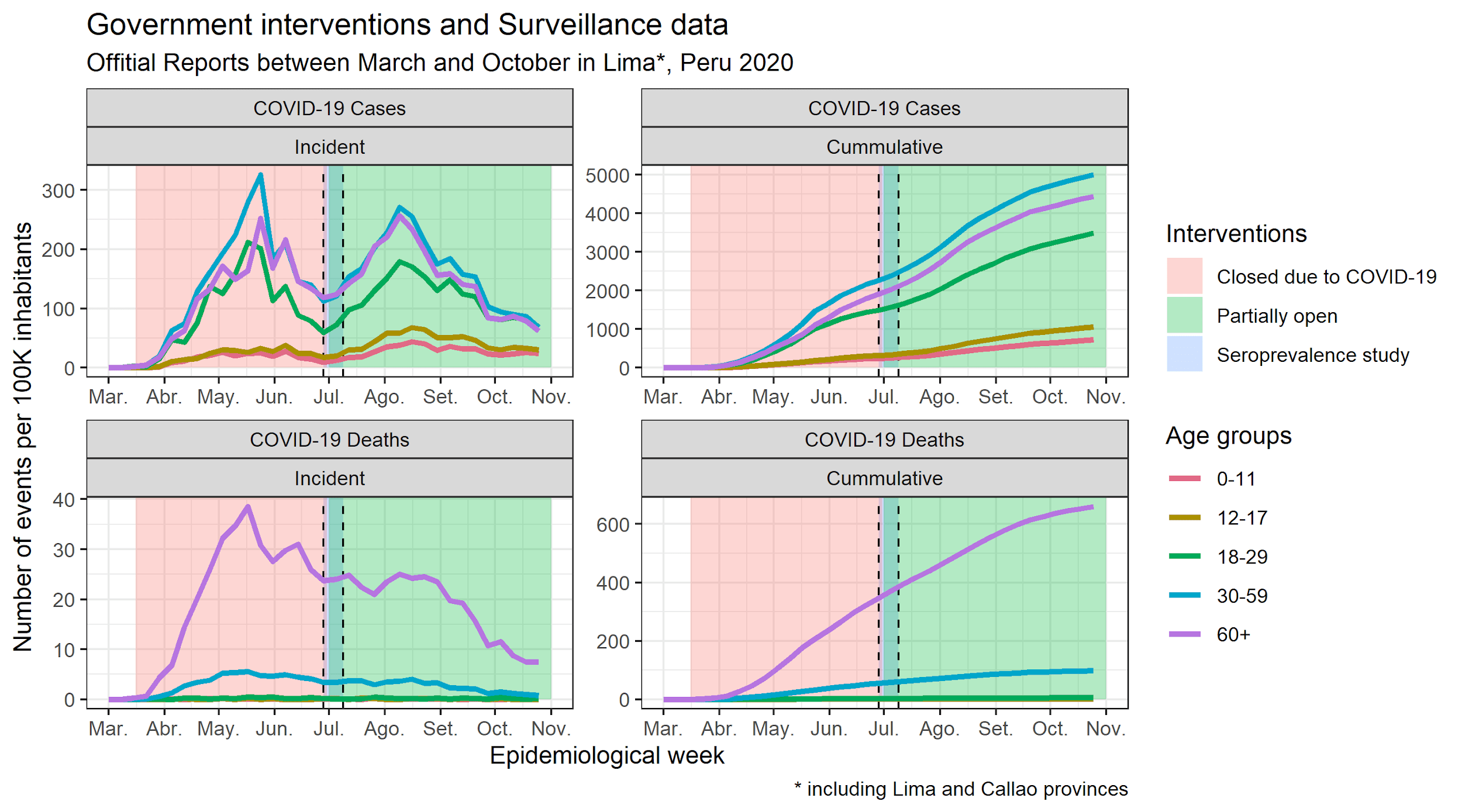
**

**Figure S3**

**QUESTIONNAIRE**

**Cuestionario ESPI- SAR-CoV-2 Lima-Callao**

Las preguntas con “ **”** es de opción única.
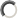


Las preguntas con “**☐”** es de opción múltiple.

Las preguntas con “______ **”** es para ser llenado.

1. **INFORMACIÓN GENERAL**

| **1. Conglomerado N.º** | _ _ _ _ _ _ _ _ _ _ _ _ _ _ _ _ _ _ _ _ _ _ _ _ _ _ _ _ _ _ |
| --- | --- |
| **2. Número de vivienda** | _ _ _ _ _ _ _ _ _ _ _ _ _ _ _ _ _ _ _ _ _ _ _ _ _ _ _ _ _ _ |
| **3. Departamento de residencia** | _ _ _ _ _ _ _ _ _ _ _ _ _ _ _ _ _ _ _ _ _ _ _ _ _ _ _ _ _ _ |
| **4. Provincia de residencia** | _ _ _ _ _ _ _ _ _ _ _ _ _ _ _ _ _ _ _ _ _ _ _ _ _ _ _ _ _ _ |
| **5. Distrito de residencia** | _ _ _ _ _ _ _ _ _ _ _ _ _ _ _ _ _ _ _ _ _ _ _ _ _ _ _ _ _ _ |
| **6. Dirección (**Ser específicos) | _ _ _ _ _ _ _ _ _ _ _ _ _ _ _ _ _ _ _ _ _ _ _ _ _ _ _ _ _ _ |
| **7a. Latitud** | (ingreso manual de coordenadas)  _ _ _ _ _ _ _ _ _ _ _ _ _ _ _ _ _ _ _ _ _ _ _ _ _ _ _ _ _ _ |
| **7b. Longitud** | (ingreso manual de coordenadas)  _ _ _ _ _ _ _ _ _ _ _ _ _ _ _ _ _ _ _ _ _ _ _ _ _ _ _ _ _ _ |
| **8. ¿Cuántas personas van a participar del estudio?** | (número de participantes)  _ _ _ _ _ _ _ _ _ _ _ _ _ _ _ _ _ _ _ _ _ _ _ _ _ _ _ _ _ _ |

Llenar Consentimientos y asentimientos informados según el número de participantes que participaran. Así mismo el tomador de muestra empezará a realizar su labor (Prueba rápida), empezando de preferencia con el primer entrevistado (jefe de hogar principal). Esto le permitirá tener un lapso de tiempo hasta obtener los resultados.

| **9. ¿Agregar miembro?** | - Si - No (En caso no hubiera más miembros que agregar, terminar la encuesta y pasar a la siguiente vivienda) |
| --- | --- |


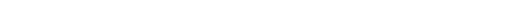


1. **INFORMACIÓN DE LA VIVIENDA**

(Solo corresponde una por vivienda, y la responderá el jefe de hogar principal, preferentemente)

| **10. Tipo de vivienda particular**  Por observación directa | - Casa independiente - Departamento en edifico - Vivienda en quinta - Vivienda en casa de vecindad (Callejón o quinta) - Local no destinado para habitación humana - Otro tipo   Especificar _ _ _ _ _ _ _ _ _ _ _ _ |
| --- | --- |
| **11. El agua que utilizan en la vivienda ¿Procede principalmente de?** | - Red pública dentro de la vivienda - Red pública fuera de la vivienda - Pilón o pileta de uso publico - Camión cisterna u otro similar - Pozo (Agua subterránea) - Otro   Especificar _ _ _ _ _ _ _ _ _ _ _ _ |
| **12. El baño o servicio higiénico que tiene la vivienda**  **¿Está conectado a?** | - Red pública de desagüe dentro de la vivienda - Red pública de desagüe fuera de la vivienda, pero dentro de la edificación - Pozo séptico, tanque séptico o biodigestor - Letrina (Con tratamiento) - Pozo ciego o negro - Otro   Especificar _ _ _ _ _ _ _ _ _ _ _ _ |
| **13. La vivienda tiene alumbrado eléctrico por red publica** | - 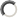 Si - 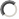 No |
| **14. ¿Cuántas habitaciones en total tiene la vivienda, sin contar el baño, la cocina, los pasadizos, ni el garaje?** | Colocar el número de dormitorios  _ _ _ _ _ |
| **15. Número de residentes habituales en este domicilio *Si hay personas que*** *NO* ***pertenecen al hogar, pero acuden de forma regular al domicilio no deben ser***  ***consideradas (Empleados, cuidadores, entre otros)*** | Colocar cuantas personas en la vivienda  _ _ _ _ _ |

A partir de la sección C de la encuesta corresponde a una entrevista individual. En caso de que se deban agregar más miembros de la vivienda se adjuntarán más formatos de entrevista individual.

1. **INFORMACIÓN PERSONAL**

| **16. NÚMERO DE HOGAR** | (Tratar que el hogar 1, sea el hogar principal)  _ _ _ _ _ _ _ _ _ _ _ _ _ _ _ _ _ _ _ _ _ _ _ _ _ _ _ _ _ | |
| --- | --- | --- |
| **17. ESTATUS DEL ENTREVISTADO** | - Presente - Hospitalizado (Solo si tiene prueba COVID positivo) | |
| **18. Tipo de documento** | - DNI - Carnet de extranjería - Pasaporte - Otros   Especificar _ _ _ _ _ _ _ _ _ _ _ _   - No tiene | |
| **19. Número de documento** | _ _ _ _ _ _ _ _ _ _ _ _ _ _ _ _ _ _ _ _ _ _ _ _ _ _ _ _ _ | |
| **20. Nombres** | _ _ _ _ _ _ _ _ _ _ _ _ _ _ _ _ _ _ _ _ _ _ _ _ _ _ _ _ _ | |
| **21. Apellido Paterno** | _ _ _ _ _ _ _ _ _ _ _ _ _ _ _ _ _ _ _ _ _ _ _ _ _ _ _ _ _ | |
| **22. Apellido Materno** | _ _ _ _ _ _ _ _ _ _ _ _ _ _ _ _ _ _ _ _ _ _ _ _ _ _ _ _ _ | |
| **23. Número de celular** | _ _ _ _ _ _ _ _ _ _ _ _ _ _ _ _ _ _ _ _ _ _ _ _ _ _ _ _ _ | |
| **24. Número fijo** | _ _ _ _ _ _ _ _ _ _ _ _ _ _ _ _ _ _ _ _ _ _ _ _ _ _ _ _ _ | |
| **25. Sexo** | - Femenino - Masculino | |
| **26. Fecha de nacimiento** | dd/mm/aaaa  _ _ / _ _ / _ _ _ _ | |
| **27a. ¿Es usted jefe del Hogar?** | - Si (Pasar a 29) - No (Pasar a 28) | |
| **En caso de no ser jefe de hogar indicar,**  **28. ¿Cuál es el parentesco con el jefe de hogar?,** | - Esposo(a) o compañero(a) - Hijo(a)/Hijastro(a) - Yerno/nuera - Nieto(a) - Padre/madre/suegro(a) - Hermano(a) - Otro pariente - Otro no pariente | |
| **29. Edad** | _ _ _ _ _ _ _ años | |
| **30. País de origen** | - Perú   Otros -> Especificar: _ _ _ _ _ _ _ _ _ _ _ _ _ _ | |
| **31. Por sus costumbres y antepasados, ¿Usted se considera?** | - ¿Quechua? - ¿Aimara? - ¿Nativo o indígena de la selva? - ¿Perteneciente o parte de otro pueblo indígena u originario? - Negro, moreno, zambo, mulato/pueblo afroperuano o afrodescendiente? - ¿Blanco? - ¿Mestizo? - ¿Otros? -> Especificar: _ _ _ _ _ _ _ _ _ _ _ _ | |
| **32. ¿Cuál fue el último nivel de estudio que aprobó?** | - Sin Nivel - Educación Inicial - Primaria incompleta - Primaria completa - Secundaria incompleta - Secundaria completa - Superior No Univ. incompleta - Superior No Univ. completa - Superior Univ. incompleta - Superior Univ. completa | |
| **33. Los últimos 14 días. ¿Trabajó para obtener un ingreso o para colaborar con la producción en el hogar?**  **Solo preguntar a mayores de 8 años** | - Si (Pasar a la 33) - No (Pasar a la 36) | |
| **34. Los últimos 14 días, ¿Cuál fue/es la ocupación principal que desempeñó?** | _ _ _ _ _ _ _ _ _ _ _ _ _ _ _ _ _ _ _ _ _ _ _ _ _ _ _ _ _  Por ejemplo: profesor de educación secundaria, abogado,  peón, vendedor ambulante de comida, etc. | |
| **35. En los últimos 14 días, ¿A qué actividad se dedicó el negocio, organismo o empresa en la que trabajo?**  **Cual fue es el rubro. Se están considerando principalmente los presentes durante la pandemia.** | - Comercio - Transporte Seguridad, FFAA, etc. - Limpieza | |
|  | - Salud (Pasar a la 35) - Cuidado de persona dependiente a domicilio - Otros - Especificar _ _ _ _ _ _ _ _ _ _ _ _ |  |
| **36. ¿Qué profesión específica en salud tiene?**  Solo para los que sean del rubro de Salud, sino pasar a 36 | - Médico - Enfermero - Obstetra - Biólogo - Tecnólogo Médico - Técnico de Enfermería - Técnico de laboratorio - Otros - Especificar _ _ _ _ _ _ _ _ _ _ _ _ |  |
| **37. Se encuentra afiliado al:** | - Seguro integral de salud (SIS) - EsSalud - Seguro de Fuerzas armadas y policiales - Seguro privado de salud - Otro seguro - Especificar _ _ _ _ _ _ _ _ _ _ _ _ - Ninguno |  |

1. **ENCUESTA EPIDEMIOLÓGICA INDIVIDUAL**

| **38. ¿Le han tomado previamente alguna prueba diagnóstica para COVID-19?** | - Si (Pasar a la 39) - No (Pasar a la 43) |
| --- | --- |
| **39. Indicar tipo de prueba** | - Prueba rápida (Pasar a la 40) - PCR (Pasar a la 41) - Ambas (Pasar a la 40) |
| **40. Resultado de la Prueba Rápida previa** | - Negativo - IgM Reactivo - IgG Reactivo - IgM e IgG Reactivo - Positivo, pero no sabe cual   En caso de ser positivo pasar a la 41, caso contrario pasar a la 42. |
| **41. Resultado de la Prueba PCR previa** | - Positivo - Negativo   En caso de ser positivo pasar a la 42, caso contrario pasar a la 43. |
| **Solo a quienes hayan tenido alguna prueba positiva**  **42. ¿Ud. realizó aislamiento por 14 días, al saberse caso de COVID-19?** | - Sí, totalmente - Sí, pero parcialmente - No - No aplica |
| **43. ¿Ha tenido contacto con alguien con sospecha o confirmación de infección por el virus del COVID- 19?** | - Sí, caso confirmado (Pasar a la 43a) - Sí, caso sospechoso (Pasar a la 43a) - No (Pasar a la 46) - Desconocido (Pasar a la 46) |
| **43a.En caso afirmativo, fecha del último contacto** | dd/mm/aaaa  _ _ / _ _ / _ _ _ _ |
| **44. Este contacto fue** | - Un miembro de su hogar - Otro miembro de la familia - Compañero de trabajo - Un amigo/a - Otros |
| **45. ¿Ud. realizó cuarentena o aislamiento en casa u otro lugar, por 14 días, al saberse contacto de un caso COVID-19?** | - Sí, totalmente - Sí, pero parcialmente - No |
| **46.Desde el 6 de marzo, ¿ha realizado algún viaje fuera de su Lima?** | - Si - No |
| **47. ¿Ha viajado en los 14 días anteriores a la fecha de inicio de síntomas?** | - Si - No |

1. **HISTORIAL DE SÍNTOMAS**

| **48. ¿Presenta actualmente algún tipo de síntoma compatible con COVID-19?** | - Si (Pasar a la 49) - No (Pasar a la 50) |
| --- | --- |
| **Solo si tiene síntomas**  **49. Indique qué síntomas tiene**  **(puede marcar más de una alternativa)** | - Fiebre ≥ 38oC - Escalofríos - Fatiga - Dolor muscular (mialgias) - Dolor de garganta - Tos - Rinorrea / congestión nasal - Dificultad para respirar (disnea/taquipnea) - Sibilancias - Dolor en el pecho - Otros síntomas respiratorios - Cefalea - Náuseas/vómitos - Dolor abdominal - Diarrea - Alteración del sentido del olfato o gusto (anosmia/disgeusia)   Otros : _ _ _ _ _ _ _ _ _ _ _ |
| **Solo si tiene síntomas 49a.Fecha de inicio de síntomas** | dd/mm/aaaa  _ _ / _ _ / _ _ _ _ |
| **50. Si no presenta síntomas actualmente. Desde el mes de marzo de 2020 a la fecha, ¿ha tenido alguno de los siguientes síntomas compatibles con COVID-19?** | - Si (Pasar a la 51) - No (Pasar a la 52) |
| **51. Indique qué síntomas tiene**  **(puede marcar más de una alternativa)** | - Fiebre ≥ 38oC - Escalofríos - Fatiga - Dolor muscular (mialgias) - Dolor de garganta - Tos - Rinorrea / congestión nasal - Dificultad para respirar (disnea/taquipnea) - Sibilancias - Dolor en el pecho - Otros síntomas respiratorios - Cefalea - Náuseas/vómitos - Dolor abdominal - Diarrea - Alteración del sentido del olfato o gusto (anosmia/disgeusia)   Otros : _ _ _ _ _ _ _ _ _ _ _ |
| **51a. Fecha de inicio de síntomas** | dd/mm/aaaa  _ _ / _ _ / _ _ _ _ |
| **52. Observación** | _ _ _ _ _ _ _ _ _ _ _ _ _ _ _ _ _ _ _ _ _ _ _ _ _ _ _ _ |

1. **BÚSQUEDA DE ATENCIÓN EN SALUD**

| **53. Desde el mes de marzo, ¿Llamó al 113 o alguno de los teléfonos brindados por las DIRIS/DIRESA, o dejo sus datos en la plataforma web del MINSA, para solicitar la prueba de tamizaje?** | - Sí y vinieron a tomarme la prueba - Sí, pero no me tomaron la prueba - No llamé ni solicité la prueba por otra plataforma |
| --- | --- |
| **54. ¿Por alguno de estos síntomas necesitó buscar atención médica en un establecimiento de salud o atención a domicilio?**  **Solo para aquellos que hayan presentado síntomas.**  **Sino pasar a la 55** | - Sí - No |

1. **PRUEBA RÁPIDA**

| **55. Procedencia de la solicitud de diagnóstico** | - Estudio de seroprevalencia (SISCOVID va tener que   agregar esta opción) |
| --- | --- |
| **56. El sujeto está presente para la prueba rápida** | - Sí (Pasar a la 56) - No (Únicamente si está hospitalizado) |
| **57. Fecha de ejecución de la Prueba Rápida** | dd/mm/aaaa  _ _ / _ _ / _ _ _ _ |
| **58. Resultado de la Prueba Rápida** | - No reactivo (Realizar hisopado, pasar a la 60) - Indeterminado (Se realizará 2da prueba, pasar a la 59) - IgM Reactivo - IgG Reactivo - IgM e IgG Reactivo |
| **59. Resultado de la Prueba Rápida 2** | - No reactivo (Realizar hisopado, pasar a la 60) - IgM Reactivo - IgG Reactivo - IgM e IgG Reactivo |
| **60. Detallar el tipo de muestra para la prueba molecular** | - Hisopado nasal o faríngeo - No fue posible |
| **61. ¿El sujeto cumple con alguna condición de riesgo?** | - Sí (Pasar a la 62) - No (Culminar) |
| **62. Especifique cuál o cuales:**  ***(puede marcar más de una alternativa)*** | - Mayor de 60 años - Hipertensión arterial - Enfermedad cardiovascular - Diabetes - Obesidad - Asma - Enfermedad pulmonar crónica - Insuficiencia renal crónica - Enfermedad o tratamiento inmunosupresor - Cancer - Embarazo o puerperio - Personal de salud - Otra condición de riesgo - Otros: _ _ _ _ _ _ _ |

**REFERENCES**

1. [Geo Perú. [GEO PERU - Georeferenced Digital Platform of the Peruvian Government] [Internet]. 2020 [cited 2020 Oct 5]. Available from:](http://paperpile.com/b/pdhLeo/KrGjN) <http://visor.geoperu.gob.pe>

2. [REUNIS. REUNIS: National Health Information Repository - Ministry of Health of Peru [Internet]. 2020 [cited 2020 Oct 5]. Available from:](http://paperpile.com/b/pdhLeo/8C8Uo) <https://www.minsa.gob.pe/reunis/index.asp>

3. [UNESCO. Education: From disruption to recovery [Internet]. 2020 [cited 2020 Oct 5]. Available from:](http://paperpile.com/b/pdhLeo/60vuH) <https://en.unesco.org/covid19/educationresponse>

4. [Valle-Campos A, Chacón H. covid19viz: Visualize COVID-19 country-level data of cases, mobility and interventions. R package version 1.0.1. https://avallecam.github.io/covid19viz/. 2020 Oct 22 [cited 2020 Oct 22]; Available from:](http://paperpile.com/b/pdhLeo/z1Bug) <https://zenodo.org/record/4118750>

5. [MINSA. [COVID-19 Positive Cases] [Internet]. 2020 [cited 2020 Oct 5]. Available from:](http://paperpile.com/b/pdhLeo/4ANsH) <https://www.datosabiertos.gob.pe/dataset/casos-positivos-por-covid-19-ministerio-de-salud-minsa>

6. [SINADEF. [SINADEF: Information on Deaths from the National Informatic System of Deaths - Ministry of Health of Peru] [internet]. 2020 [cited 2020 Oct 5]. Available from:](http://paperpile.com/b/pdhLeo/qHVj6) <https://www.datosabiertos.gob.pe/dataset/informaci%C3%B3n-de-fallecidos-del-sistema-inform%C3%A1tico-nacional-de-defunciones-sinadef-ministerio>

7. [MINSA. [COVID-19 Deaths] [Internet]. 2020 [cited 2020 Oct 5]. Available from:](http://paperpile.com/b/pdhLeo/VYGJl) <https://www.datosabiertos.gob.pe/dataset/fallecidos-por-covid-19-ministerio-de-salud-minsa>

8. [Incio J. covidPeru: Funciones para procesar información sobre COVID-19 en Perú [Internet]. 2020 [cited 2020 Oct 5]. Available from:](http://paperpile.com/b/pdhLeo/olKXX) <https://github.com/jincio/covidPeru>

9. [INEI. [Lima Metropolitan Area: Socio-Demographic Profile] [Internet]. 1996 [cited 2020 Oct 6]. Available from:](http://paperpile.com/b/pdhLeo/gNgmY) <http://proyectos.inei.gob.pe/web/biblioineipub/bancopub/Est/LIb0002/mapaprov.htm>

10. [FDA. openFDA: Independent Evaluations of COVID-19 Serological Tests [Internet]. 2020 [cited 2020 Oct 16]. Available from:](http://paperpile.com/b/pdhLeo/T6Cz1) <https://open.fda.gov/apis/device/covid19serology/>

11. [FIND. SARS-CoV-2 diagnostics: performance data [Internet]. 2020 [cited 2020 Oct 6]. Available from:](http://paperpile.com/b/pdhLeo/uVukz) <https://www.finddx.org/covid-19/dx-data/>

12. [Healgen. Product Overview: COVID-19 IgG/IgM Rapid Test Cassette (Whole Blood/Serum/Plasma) [Internet]. 2020 [cited 2020 Oct 16]. Available from:](http://paperpile.com/b/pdhLeo/GFZQa) <http://www.orientgene.com/asp-en/product/list.aspx?category_id=1094&product_id=8398>

13. [Vidal-Anzardo M, Solis G, Solari L, Minaya G, Ayala-Quintanilla B, Astete-Cornejo J, et al. Evaluation of a rapid serological test for detection of IgM and igG antibodies against SARS-CoV-2 under field conditions. Rev Peru Med Exp Salud Publica. 2020 Apr;37(2):203–9.](http://paperpile.com/b/pdhLeo/HLgrM)

14. [Pollán M, Pérez-Gómez B, Pastor-Barriuso R, Oteo J, Hernán MA, Pérez-Olmeda M, et al. Prevalence of SARS-CoV-2 in Spain (ENE-COVID): a nationwide, population-based seroepidemiological study. Lancet [Internet]. 2020 Jul 3; Available from:](http://paperpile.com/b/pdhLeo/qi5vx) <http://dx.doi.org/10.1016/S0140-6736(20)31483-5>

15. [INEI. Censos Nacionales 2017 [Internet]. 2018 [cited 2020 Nov 18]. Available from:](http://paperpile.com/b/pdhLeo/JGaiS) <http://censo2017.inei.gob.pe/>

16. [Freedman G, Schneider B. srvyr: “dplyr”-Like Syntax for Summary Statistics of Survey Data. http://gdfe.co/srvyr [Internet]. 2020 [cited 2020 Oct 6]. Available from:](http://paperpile.com/b/pdhLeo/VShmi) <https://github.com/gergness/srvyr>

17. [Larremore DB, Fosdick BK, Zhang S, Grad YH. Jointly modeling prevalence, sensitivity and specificity for optimal sample allocation [internet]. 2020. Available from:](http://paperpile.com/b/pdhLeo/QIB6k) <http://dx.doi.org/10.1101/2020.05.23.112649>

18. [Valle-Campos A. serosurvey: Serological Survey Analysis For Prevalence Estimation Under Misclassification. R package version 1.0.1. https://avallecam.github.io/serosurvey/. 2020 Oct 22 [cited 2020 Oct 22]; Available from:](http://paperpile.com/b/pdhLeo/SsrlK) <https://zenodo.org/record/4118710>

19. [Lumley T. Complex Surveys: A Guide to Analysis Using R. John Wiley & Sons; 2011. 296 p.](http://paperpile.com/b/pdhLeo/sufYe)

20. [Valle-Campos A. epitidy: R package to summarise, tidy up model outputs and generate raw tables in a Epidemiologist-way. R package version 1.0. 2020 Oct 4 [cited 2020 Nov 18]; Available from:](http://paperpile.com/b/pdhLeo/grU86) <https://zenodo.org/record/4065078>
